# Methyl-lysine readers PHF20 and PHF20L1 define two distinct gene expression–regulating NSL complexes

**DOI:** 10.1016/j.jbc.2022.101588

**Published:** 2022-01-14

**Authors:** Hieu T. Van, Peter R. Harkins, Avni Patel, Abhinav K. Jain, Yue Lu, Mark T. Bedford, Margarida A. Santos

**Affiliations:** 1 Department of Epigenetics and Molecular Carcinogenesis, The University of Texas MD Anderson Cancer Center, Houston, Texas, USA; 2 Center for Cancer Epigenetics, The University of Texas MD Anderson Cancer Center, Houston, Texas, USA; 3 Graduate Program in Genetics & Epigenetics, The University of Texas MD Anderson Cancer Center UTHealth Graduate School of Biomedical Sciences, Houston, Texas, USA

**Keywords:** transcriptional regulation, epigenetics, histone acetylation, lysine methylation, housekeeping genes, ACN, acetonitrile, ChIP-Seq, chromatin immunoprecipitation sequencing, CPRIT, Cancer Prevention Research Institute of Texas, DNMT1, DNA (cytosine-5)-methyltransferase 1, Dox, doxycycline, FBS, fetal bovine serum, FL, full-length, HA, hemagglutinin, IgG, immunoglobulin G, IP, immunoprecipitation, KD, knockdown, MEF, mouse embryonic fibroblast, MLB, mild lysis buffer, MS, mass spectrometry, MSL, male-specific lethal, NSL, nonspecific lethal, PHF20, plant homeodomain finger protein 20, PHF20L1, PHF20-like protein 1, PhI, phosphatase inhibitor, PI, protease inhibitor, pRb, phosphorylated retinoblastoma tumor suppressor protein, P/S, penicillin–streptomycin, qPCR, quantitative PCR, TBS, Tris-buffered saline, TEAB, triethylammonium bicarbonate, TES, transcription end site, TSS, transcriptional starting site, WB, Western blot, WDR5, WD repeat-containing protein 5

## Abstract

The methyl-lysine readers plant homeodomain finger protein 20 (PHF20) and its homolog PHF20-like protein 1 (PHF20L1) are known components of the nonspecific lethal (NSL) complex that regulates gene expression through its histone acetyltransferase activity. In the current model, both PHF homologs coexist in the same NSL complex, although this was not formally tested; nor have the functions of PHF20 and PHF20L1 regarding NSL complex integrity and transcriptional regulation been investigated. Here, we perform an in-depth biochemical and functional characterization of PHF20 and PHF20L1 in the context of the NSL complex. Using mass spectrometry, genome-wide chromatin analysis, and protein-domain mapping, we identify the existence of two distinct NSL complexes that exclusively contain either PHF20 or PHF20L1. We show that the C-terminal domains of PHF20 and PHF20L1 are essential for complex formation with NSL, and the Tudor 2 domains are required for chromatin binding. The genome-wide chromatin landscape of PHF20–PHF20L1 shows that these proteins bind mostly to the same genomic regions, at promoters of highly expressed/housekeeping genes. Yet, deletion of PHF20 and PHF20L1 does not abrogate gene expression or impact the recruitment of the NSL complex to those target gene promoters, suggesting the existence of an alternative mechanism that compensates for the transcription of genes whose sustained expression is important for critical cellular functions. This work shifts the current paradigm and lays the foundation for studies on the differential roles of PHF20 and PHF20L1 in regulating NSL complex activity in physiological and diseases states.

Transcription is a complex process that involves several layers of fine-tuned regulation ranging from chromatin remodeling and histone modifications to RNA processing and export (1). Well-known systems that allow for studies aimed at defining the relationship between transcriptional activation and chromatin structure are the inactive female X chromosome in mammals and the hyperactive male X chromosome in *Drosophila*, which ensure equalization of X-linked gene expression in the different sexes. A major player in the dosage compensation complex in flies is the histone acetyltransferase KAT8, also known as MOF (males absent on the first) or MYST1 (2, 3). KAT8 is part of the male-specific lethal (MSL) complex in *Drosophila*, where it is essential for sex chromosome dosage compensation in males (3, 4, 5). The MSL complex binds to the male X chromosome, where it catalyzes H4K16ac leading to transcription activation (3, 6, 7). KAT8 also serves as the catalytic subunit of the nonspecific lethal (NSL) complex (8). The NSL complex has been found to localize at promoters of constitutively active housekeeping genes (9, 10, 11). Until recently, the prevalent thought in the literature was that NSL-directed phenotypes primarily resulted from H4K16ac catalytic activity (8, 12); however, some *in vitro* studies show that the NSL complex can also acetylate H4K5 and H4K8 (13, 14). A recent study has definitively shown that KAT8 catalyzes H4K5 and H4K8 acetylation *in vivo* as part of the NSL complex, as opposed to catalyzing H4K16ac as part of the MSL complex; further, this study shows that the NSL complex is essential for cell survival, whereas the MSL complex is not (15).

Among the proteins found in the NSL complex, there are two related effector molecules that read lysine methylation residues in histones and nonhistone proteins: plant homeodomain finger protein 20 (PHF20) and PHF20-like protein 1 (PHF20L1) (13, 16, 17). PHF20 has two N-terminal Tudor domains, one AT hook domain, one zinc finger domain, and one C-terminal PHD finger domain (18). PHF20 directly binds to p53 dimethylated at K370 or K382 through its dimerized second Tudor, which greatly enhances binding of p53 to PHF20. This interaction leads to stabilization and activation of p53, and it contributes to the upregulation of p53 upon DNA damage (19, 20). Furthermore, *in vivo* and *in vitro* studies demonstrate that PHF20 transcriptionally regulates p53 in an Akt-dependent manner and promotes NF-κB transcriptional activity (21, 22). The PHD finger domain can recognize H3K4me2 residues and affects its methylation along with the mixed lineage leukemia 1–lysine methyltransferase complex (20, 23). PHF20 deficiency in mice results in perinatal lethality and various defects in the skeletal and hematopoietic systems; intriguingly, the loss of PHF20 results in decreased gene expression, but the levels of H4K16ac remain unaltered (24). This agrees with the recent data demonstrating that the main catalytic activity of the NSL complex is not H4K16ac (15). PHF20 was first identified as an antigen in glioblastoma patients and is highly expressed in different tumors, with potential roles in the development and progression of different cancers, such as glioma, adenocarcinomas, and lung cancer (25, 26, 27, 28).

Like PHF20, PHF20L1 contains two Tudor domains. However, the Tudor domains of PHF20L1 were shown to interact with monomethylated lysine residues in H3K4 and H4K20 (29) and more recently with H3K27 dimethyl residues (30). In addition to histones, PHF20L1 binds to monomethylated K142 of DNA (cytosine-5)-methyltransferase 1 (DNMT1) (31) that is involved in the regulation of DNMT1 proteosome degradation (32, 33). PHF20L1 was also shown to read monomethylated K810 of phosphorylated retinoblastoma tumor suppressor protein (pRb), which allows pRb activity to be effectively integrated with the DNA damage response (34). In addition, PHF20L1 has also been shown to protect SOX2 from methylation-dependent proteolysis (35, 36). Although PHF20L1-deficient mice survive, the loss of PHF20L1 leads to delayed growth in both sexes and abnormal mammary gland development in female mice (30). Aberrations in the *PHF20L1* gene are highly correlated with various cancers, such as ovarian and breast cancer (37, 38). For example, in breast cancer, depletion of PHF20L1 suppresses cancer growth (30, 35).

Despite their similarities regarding protein domain structures and the fact that both PHF20 and PHF20L1 complex with NSL, it remains unclear whether they can functionally compensate for each other and how each contributes to the integrity of the NSL complex.

In this study, we identify the existence of two distinct NSL complexes that exclusively contain either PHF20 or PHF20L1; the two PHF homologs do not complex together in the same NSL species. The genome-wide landscape of PHF20–PHF20L1 binding to chromatin shows that they bind mostly to the same genomic regions, although a subset of target genes is differentially bound by PHF20 only. Moreover, we define which domains in each protein are required for the interaction with NSL and the binding to the specific chromatin locations. Both PHF20 and PHF20L1 bind to highly expressed genes/housekeeping genes; yet deletion of either or both does not abrogate gene expression at the identified target genes nor the recruitment of NSL to the promoters of those genes, posing the possibility of an alternative compensatory mechanism that sustains the transcription of genes required for crucial cellular functions.

## Results

### Identification of two distinct NSL complexes that exclusively contain PHF20 or PHF20L1

**Figure 1:**
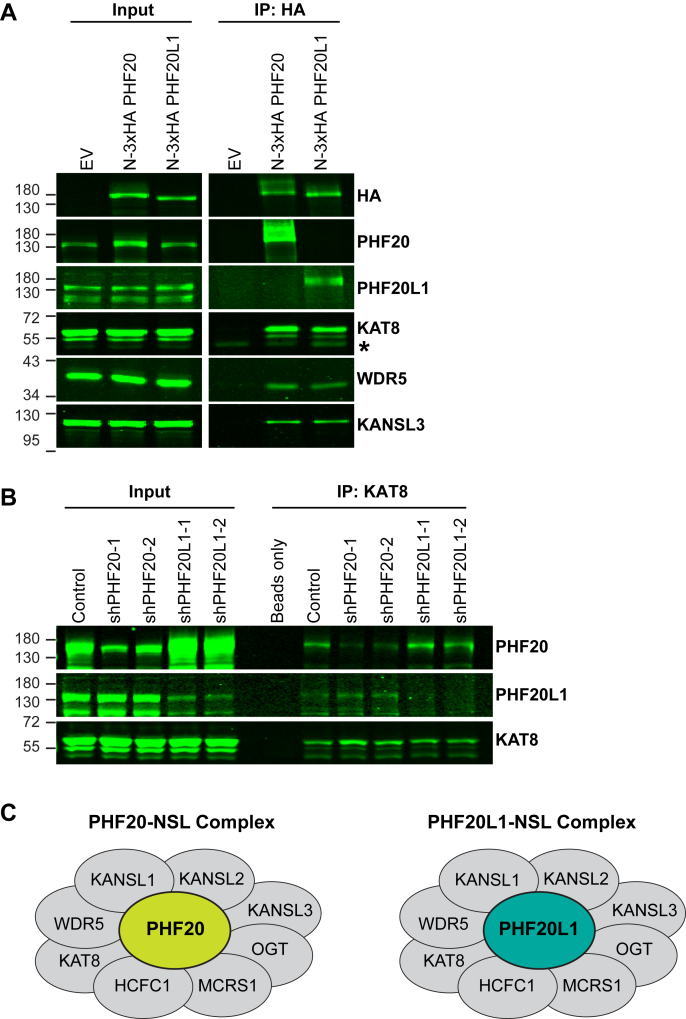
**PHF20 and PHF20L1 exclusively and independently interact with the NSL complex.***A*, Western blotting (WB) results of HA immunoprecipitation (IP) from U2OS overexpressing empty vector (EV), 3xHA PHF20, or 3xHA PHF20L1. Respective WB antibodies used are shown. *B*, WB results of KAT8 IP from U2OS carrying control vector (Control), shRNAs for *PHF20* (shPHF20-1 and shPHF20-2), or shRNAs for *PHF20L1* (shPHF20L1-1 and shPHF20L1-2). Respective WB antibodies used are shown. *C*, the composition of two distinct NSL complexes: PHF20-NSL and PHF20L1-NSL. ∗ denotes signal from heavy-chain immunoglobulin G (IgG). At least two independent IP and WB analyses were done to confirm the results. HA, hemagglutinin; NSL, nonspecific lethal; PHF20, plant homeodomain finger protein 20; PHF20L1, PHF20-like protein 1.

PHF20 and PHF20L1 have long been known to complex with NSL, and the assumed models depict both proteins within the same NSL complex (13, 16, 17). However, whether these two homologs are present together in the same NSL complex or exclusively in distinct NSLs was, to our knowledge, never investigated. To answer this question, we overexpressed either FLAG-tagged PHF20 (FLAG-PHF20) or FLAG-tagged PHF20L1 (FLAG-PHF20L1) in U2OS cells and performed immunoprecipitation (IP) against FLAG. The immunoprecipitated products were then analyzed by mass spectrometry (MS). As shown in Table 1, our IP–MS results show that both PHF20 and PHF20L1 are associated with KAT8 and all known subunits of the NSL complex (KAT8-associated nonspecific lethal proteins 1 to 3 [KANSL1–3], UDP-*N*-acetylglucosaminepeptide *N-*acetylglucosaminyltransferase 110 kDa subunit, host cell factor 1, WD repeat-containing protein 5 (WDR5), and microspherule protein 1). However, PHF20 and PHF20L1 are not associated with each other, indicating that two different NSL complexes might exist: one with PHF20 and one with PHF20L1. We further confirmed the MS data by performing IP for hemagglutinin (HA) in 3xHA-PHF20 and 3xHA-PHF20L1 U2OS cells (Figs. S1*A* and S10*A*); Western blot (WB) for PHF20, PHF20L1, and other subunits of the NSL complex such as KAT8, WDR5, and KANSL3 shows that PHF20 and PHF20L1 associate separately with the NSL complex (Figs. 1*A* and S9*A*). Furthermore, when we ectopically expressed FLAG-tagged PHF20 in cells expressing exogeneous 3xHA-tagged PHF20L1 and performed an IP for FLAG, we pulled down PHF20 but not PHF20L1 (Figs. S1*B* and S10*B*), showing that these two proteins do not interact with each other. These experiments were performed with a HA tag inserted in the N-terminal domain. We also tagged the C-terminal domains (Figs. S1*A* and S10*A*), and the cellular localization of PHF20 and PHF20L1 as well as the integrity of the complex remained unchanged (Figs. S1, *C* and *D* and S10, *C* and *D*). Furthermore, to exclude an artifact of the overexpression, we overexpressed detected proteins in cells with knockdown (KD, by shRNA) of *PHF20* or *PHF20L1*. Once again, we observed that the integrity of the complex remains the same (Figs. S2 and S10, *E–G*).

**Table 1:** Subunits of NSL complex were identified by MS analysis of FLAG immunoprecipitated products from U2OS overexpressing FLAG-tagged PHF20 or PHF20L1

Identified proteins	Alternative IDs	IP: FLAG PHF20			IP: FLAG PHF20L1		
		Unique peptides	Coverage (%)	Identification probability (%)	Unique peptides	Coverage (%)	Identification probability (%)
PHD finger protein 20	PHF20	17	26	100	—	—	—
PHD finger protein 20like 1	PHF20L1	—	—	—	16	23	100
KAT8 regulatory NSL complex subunit 1	KANSL1	21	31	100	9	14	100
KAT8 regulatory NSL complex subunit 2	KANSL2	5	15	100	2	9	100
KAT8 regulatory NSL complex subunit 3	KANSL3	19	41	100	10	20	100
Histone acetyltransferase KAT8	KAT8	5	17	100	1	4	98
WD repeat-containing protein 5	WDR5	12	60	100	5	27	100
Host cell factor 1	HCFC1	21	20	100	13	11	100
UDP-*N*-acetylglucosaminepeptide *N*-acetylglucosaminyltransferase 110 kDa subunit	OGT	8	12	100	3	6	100
Microspherule protein 1	MCRS1	10	29	100	7	25	100

To further probe the independent association of either PHF homolog with NSL (Figs. S3*A* and S10*H*), we performed an IP for the endogenous KAT8 protein in the PHF20 or PHF20L1 KD cells mentioned previously (Figs. 1*B* and S9*B*). We could detect NSL proteins such as KAT8 in both *PHF20* or *PHF20L1* KD cells, as well as the PHF homolog that was not knocked down in each line, thus concluding that PHF20 and PHF20L1 independently associate with NSL (Fig. 1*C*).

### Chromatin landscape of PHF20 and PHF20L1 binding genome wide

Our aforementioned data show the existence of two distinct NSL complexes that differ in the presence of either PHF20 or PHF20L1 and thus raise the question of whether NSL activity is dependent on one or both PHF homologs.

**Figure 2:**
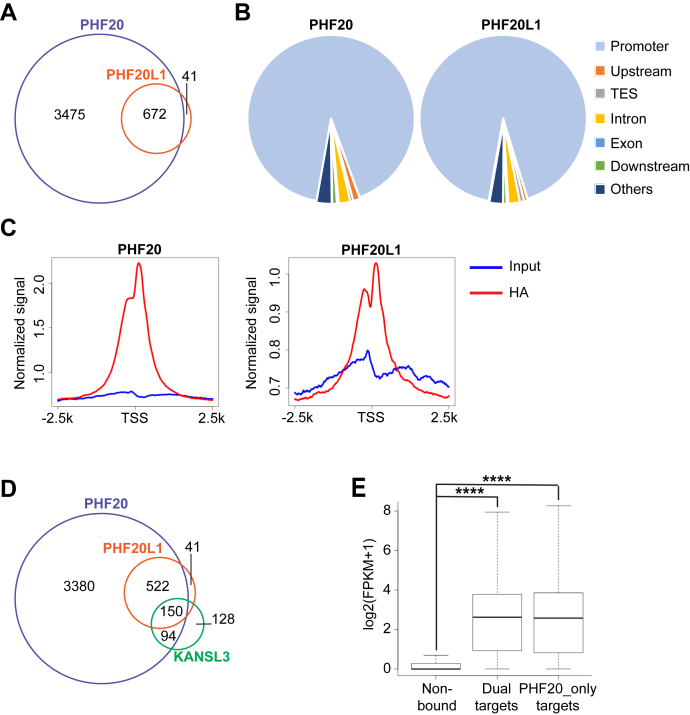
**PHF20 and PHF20L1 bind to the promoters of highly expressed genes.***A*, Venn diagram indicating the numbers and the overlap of 3xHA PHF20 and 3xHA PHF20L1 ChIP-Seq peaks. *B*, pie chart of the genomic distribution of 3xHA PHF20-binding (*left*) and 3xHA PHF20L1-binding sites. *C*, average normalized input and HA ChIP-Seq profiles of 3xHA PHF20 (*left*) and 3xHA PHF200L1 (*right*) across transcription start sites (TSSs). *D*, Venn diagram indicating the numbers and overlap of 3xHA PHF20, 3xHA PHF20L1, and KANSL3 ChIP-Seq peaks. *E*, boxplot showing the expression of genes that do not have PHF20 or PHF20L1 binding to their promoters (nonbound), genes that have both PHF20 and PHF20L1 binding to their promoters (dual targets), or genes that have only PHF20 binding to their promoters (PHF20_only targets) in U2OS cells. ∗∗∗∗*p* (by two-sided *t* test) <0.0001. ChIP-Seq, chromatin immunoprecipitation sequencing; HA, hemagglutinin; PHF20, plant homeodomain finger protein 20; PHF20L1, PHF20-like protein 1.

We first defined the chromatin landscape of PHF20 and PHF20L1 binding genome wide. To accomplish this, we performed chromatin IP followed by sequencing (chromatin immunoprecipitation sequencing [ChIP-Seq]) with an antibody against HA in U2OS cells ectopically expressing HA-tagged PHF20 or HA-tagged PHF20L1 (the cells were GFP sorted for HA enrichment right before proceeding with the library preparation; Figs. S3*B* and S10*I*). As depicted in the Venn diagram of Figure 2*A*, most of the genomic regions bound by PHF20L1 are also bound by PHF20 (672); PHF20 however binds uniquely to approximately 3500 genomic regions. All PHF20 and/or PHF20L1 binding regions were mostly mapped at gene promoters (Fig. 2, *B* and *C*).

A recent study showed that the NSL complex stimulates transcription initiation at promoters of housekeeping genes and defines the genomic targets of KANSL3, an exclusive subunit of the NSL complex (15). We observed that most of those genes are also bound by both PHF20 and/or PHF20L1 in our dataset (Fig. 2*D*). Moreover, using a publicly available RNA-Seq dataset of U2OS cells, we observed that PHF20-bound and PHF20L1-bound regions are overall enriched for genes that have high levels of gene expression (Fig. 2*E*). These data suggest that PHF20 and PHF20L1 participate in the transcription regulation of genes whose sustained expression is important for critical cellular functions.

### Complexing of either PHF20 or PHF20L1 with NSL requires their C-terminal domains

**Figure 3:**
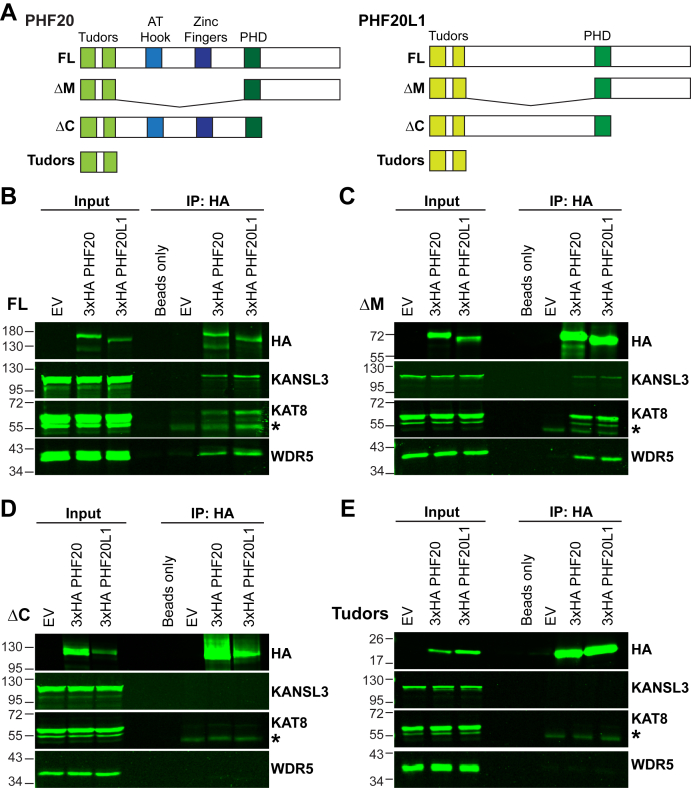
**Complexing of either PHF20 or PHF20L1 with the NSL complex requires their C termini.***A*, schematic representation of 3xHA full-length (FL) and various truncated versions (ΔM, ΔC, or Tudors) of PHF20 and PHF20L1. *B*–*E*, Western blotting (WB) results of HA immunoprecipitation (IP) from U2OS overexpressing empty vector (EV) or different versions of 3xHA PHF20 and 3xHA PHF20L1: FL (*B*), ΔM (*C*), ΔC (*D*), or Tudors (*E*). Respective WB antibodies used are shown. ∗ denotes signal from heavy-chain immunoglobulin G (IgG). At least two independent IP and WB analyses were done to confirm the results. HA, hemagglutinin; NSL, nonspecific lethal; PHF20, plant homeodomain finger protein 20; PHF20L1, PHF20-like protein 1.

Although previous studies have shown a role for PHF20 (19, 20, 21, 22, 23, 24) and PHF20L1 (34) in transcription regulation, to our knowledge, there are no studies that directly address those regulatory functions in the context of NSL except by indirect measurement of H4K16ac levels. To map the domains that are required for PHF20 or PHF20L1 to independently complex with NSL, we generated three HA-tagged truncated versions of either PHF20 or PHF20L1 (Figs. 3*A* and S4*A*). One version lacks the region between the Tudor domains and the PHD domain (ΔM); another lacks the C-terminal domain (ΔC), and the third truncated version contains only the Tudor domains (Tudors). We overexpressed these HA-tagged proteins in U2OS cells (Figs. S4*A* and S10*J*), and subcellular fractionation analysis shows that these truncated versions distribute similarly to the full-length (FL) PHF20 or PHF20L1 (Figs. S4*B* and S10*K*). We then performed a pull down for HA. As shown in Figures 3*B* and S9*C*, when we overexpressed FL PHF20 or PHF20L1, we were able to detect the NSL components KAT8, KANSL3, and WDR5. The same was true for the versions that lack the region between the Tudor domains and the PHD finger (ΔM) in both proteins (Figs. 3*C* and S9*D*). However, with the HA-tagged C-terminal truncated proteins, we were not able to pull down the NSL complex members as aforementioned (Figs. 3*D* and S9*E*); consistent with this observation, the Tudor domain–only versions also did not complex with NSL members (Figs. 3*E* and S9*F*). Taken together, these data show that the C-terminal domain is essential for PHF20 and PHF20L1 to complex with NSL.

### The Tudor2 domains of PHF20 and PHF20L1 are required for chromatin binding

**Figure 4:**
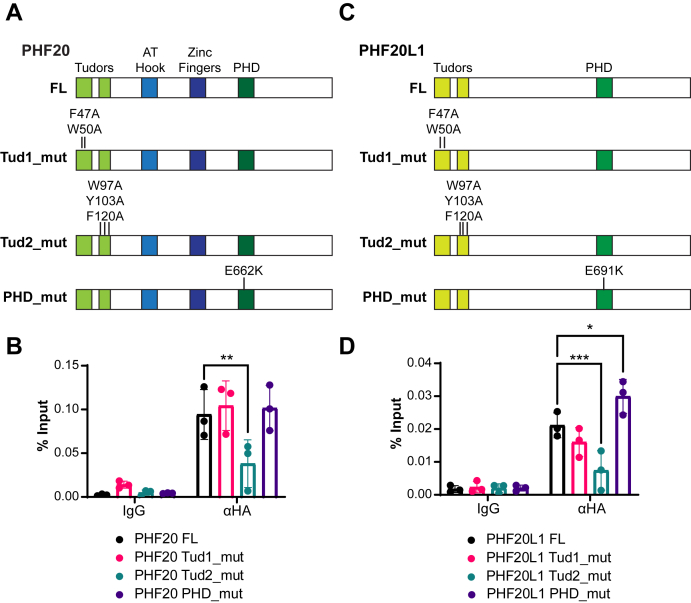
**The Tudor 2 domains of PHF20 and PHF20L1 are required for their chromatin binding.***A* and *C*, schematic diagram of 3xHA full-length (FL) and mutated versions of PHF20 (*A*) or PHF20L1 (*C*): Tudor 1 mutant (Tud1_mut), Tudor 2 mutant (Tud2_mut), and PHD mutant (PHD_mut), with the introduced mutations noted above the respective domains. *B* and *D*, ChIP–qPCR analysis of 3xHA full length (FL) or mutants of PHF20 (*B*) or PHF20L1 (*D*) at *NAGPA* transcription start site (TSS). Data represent mean ± SD, n = 3 separate ChIP–qPCR experiments from three biological replicates. ∗*p* ≤ 0.05, ∗∗*p* ≤ 0.01; ∗∗∗*p* ≤ 0.001. ChIP, chromatin immunoprecipitation; HA, hemagglutinin; PHF20, plant homeodomain finger protein 20; PHF20L1, PHF20-like protein 1; qPCR, quantitative PCR.

Previous studies in the literature reported that the Tudor2 of PHF20 binds to dimethylated estrogen receptor α (23) as well as H3K4me2 (20) *in vitro*. To define which domain of PHF20 is required for its binding to chromatin, we generated a set of HA-tagged PHF20 proteins mutated in residues shown to be important for each domain's function (18, 19, 20): one with mutations in two residues of the aromatic cage of the Tudor domain 1 (F47A and W50A) called Tud1_mut; another with mutations in three residues of the aromatic cage of the Tudor domain 2 (W97A, Y103A, and F120A) called Tud2_mut, and finally, a PHF20 protein with a mutation in the PHD domain (E662K) that we called PHD_mut (Figs. 4*A*, S5*A* and S10*L*). We overexpressed these constructs in U2OS cells and performed ChIP for HA followed by RT—quantitative PCR (qPCR) with primers spanning a region within the transcriptional starting site (TSS) of *NAGPA*, a gene that was bound by HA-PHF20 in the ChIP-Seq data (Fig. S5*B*). As shown in Figure 4*B*, FL PHF20 was detected as expected in the promoter of *NAGPA* (here, we used the N-terminal tag, please see Fig. S5*C* with similar results for the C-terminal tag); the same was observed for the PHD_mut. However, Tud2_mut PHF20 binding to chromatin was significantly reduced. We conclude that PHF20 binds to chromatin through its Tudor 2 domain.

The Tudor1 of PHF20L1 has been shown to recognize monomethylated DNMT1 (39) as well as methylated Rb (34). To define the PHF20L1 domains required for its binding to chromatin, we proceeded with the generation of HA-tagged PHF20L1 mutated in the aromatic cages (18) of the first (F47A and W50A) and second (W97A, Y103A, and F120A) Tudor domains as well as in the PHD domain (E691K) (Figs. 4*C* and S5*A*). ChIP followed by qPCR in the TSS of *NAGPA* (here, we used the N-terminal tag, please see Fig. S5*D* with similar results for the C-terminal tag), which also showed binding by PHF20L1 in our ChIP-Seq dataset (Fig. S5*B*), revealed that mutation of the Tudor 2 domain significantly reduces the binding of PHF20L1 to chromatin (Fig. 4*D*). Thus, we mapped the domains in PHF20 and PHF20L1 that are required for their binding to chromatin to the Tudor 2 domains.

### Roles of PHF20 and PHF20L1 in transcription regulation

**Figure 5:**
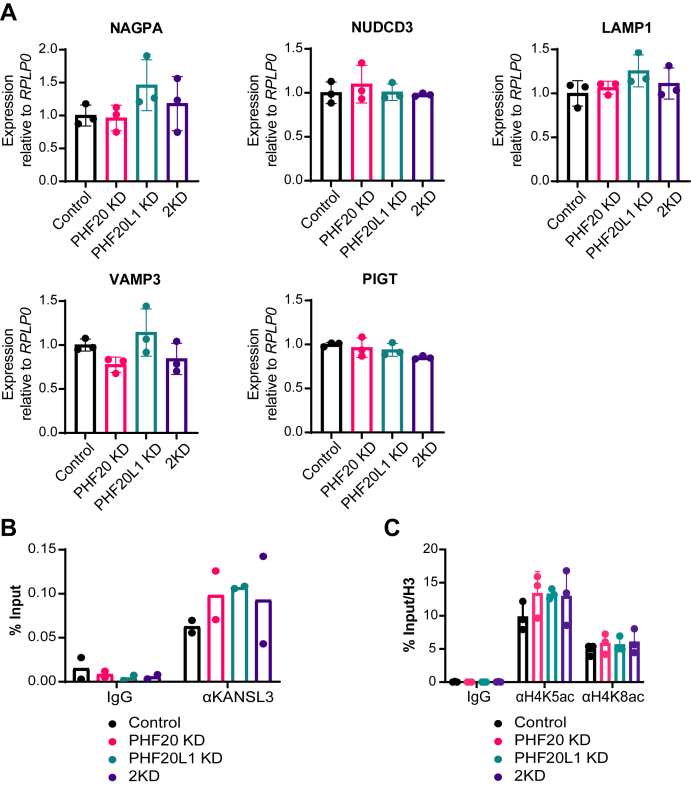
**PHF20 and PHF20L1 are dispensable for the expression of the target genes.***A*, RT–qPCR analysis of transcriptional changes in U2OS carrying control vectors (Control), shRNA for *PHF20* (PHF20 KD), shRNA for *PHF20L1* (PHF20L1 KD), or shRNAs for both *PHF20* and *PHF20L1* (2KD). *B* and *C*, ChIP–qPCR analysis of KANSL3 (*B*) and histone marks: H4K5ac and H4K8ac (*C*) at *NAGPA* transcription start site (TSS) in U2OS carrying control vectors (Control), shRNA for *PHF20* (PHF20 KD), shRNAs for *PHF20L1* (PHF20L1 KD), or shRNAs for both *PHF20* and *PHF20L1* (2KD). Data represent mean ± SD. For RT–qPCR, n = 3 from three biological replicates; for ChIP–qPCR, n = 2 (*B*) or 3 (*C*) separate ChIP–qPCR experiments from two (*B*) or three (*C*) biological replicates, respectively. ChIP, chromatin immunoprecipitation; KD, knockdown; PHF20, plant homeodomain finger protein 20; PHF20L1, PHF20-like protein 1; qPCR, quantitative PCR.

To define the role of PHF20 and PHF20L1 in transcription regulation, we chose five genes bound by PHF20 and/or PHF20L1 in our ChIP-Seq dataset: NAGPA, NUDCD3, LAMP1, PIGT, and VAMP3 (Figs. S5*B* and S6*C*). We infected U2OS cells that carry either control vector or shRNA for *PHF20L1* with an inducible shRNA for *PHF20* or its corresponding control. In this system, once the cells were induced with doxycycline (Dox), we obtained cells that have a reduction in expression of either PHF20 or PHF20L1 (PHF20 KD and PHF20L1 KD, respectively) and of both proteins (2KD; Figs. S6, *A* and *B* and S10*M*). We observed that the expression of the aforementioned PHF20-bound and/or PHF20L1-bound genes was not affected in PHF20, PHF20L1, or PHF20–PHF20L1 KD cells when compared with controls (Fig. 5*A*). Despite the fact that our KD efficiency is high (Fig. S6, *A* and *B*), we tested gene expression in mouse embryonic fibroblasts (MEFs) from PHF20 knockout animals (24). The expression of the corresponding mouse genes shown to be bound by PHF20 and or PHF20L1 in aforementioned human cells remained unchanged as compared with the wildtype cells (Figs. S7 and S10*N*). We conclude that although PHF20 and/or PHF20L1 bind to specific gene promoter regions, those genes continue to be transcribed in the absence of one or both PHF proteins.

Both PHF20 and PHF20L1 associate with the NSL complex ((13, 16, 17) and our data in Fig. 1). To test whether PHF20 and/or PHF20L1 are essential to target NSL to its specific binding locations, we performed ChIP followed by qPCR (ChIP–qPCR) using primers flanking sequences within the proximal promoter region of *NAGPA* (that showed PHF20 and PHF20L1 binding in Fig. S5*B*) with an antibody against KANSL3. As shown in Figure 5*B*, we could detect KANSL3 binding in control cells; moreover, this binding was not significantly reduced in the absence of either or both PHFs. The same was observed for the localization of H4K5ac and H4K8ac, the two major marks shown to be deposited by KANSL3 in recent studies (15) (Fig. 5*C*). Taken together, our data show that transcription at promoters bound by PHF20 and/or PHF20L1 continues to take place in the absence of one or both PHFs. This is consistent with the observed binding of NSL to the same regions even when these methyl-lysine readers are absent.

## Discussion

The MOF–NSL complex plays a crucial role in transcriptional regulation from flies to humans. However, except for KAT8, the catalytic component of the complex, little is known regarding the specific functions of the other components. By performing an in-depth biochemical and functional characterization, we now define the contributions of the two methyl-lysine reader homologs PHF20 and PHF20L1 to the NSL complex.

We unexpectedly find that two distinct NSL species exist; one containing PHF20 and the other containing PHF20L1; the two PHF homologs do not coexist in the same complex. This finding shifts the current paradigm that depicts PHF20 and PHF20L1 in the same NSL species and has potential important implications for a differential role of PHF20 and PHF20L1 in regulating NSL functions both in physiological conditions and in disease, such as cancer, where both PHFs have been shown to be altered (25, 26, 27, 28, 37, 38). In addition, we map the C terminus of PHF20 and PHF20L1 as the domain responsible for complexing with other NSL components.

When PHF20 was deleted in mice, gene expression changes were detected; interestingly, the levels of H4K16 acetylation were not dramatically changed (24). A study recently published by the Helin group showing that NSL predominantly catalyzes acetylation of H4K5 and H4K8 but not H4K16 (15) sheds light on the former since our data confirm that PHF20 does complex with NSL.

The genome-wide landscape of PHF20 and PHF20L1 binding reveals that these methyl-lysine readers bind mostly to gene promoters of highly expressing genes, including those bound by NSL and enriched for housekeeping genes. However, deletion of one or both does impact neither transcription of those genes nor recruitment of NSL. Different scenarios are possible here: (1) PHF20 and PHF20L1 are not the NSL components that target the complex to chromatin; (2) a backup system exists that compensates for the loss of PHF20 and/or PHF20L1 in recruiting NSL to the chromatin. The other two members of the NSL complex with chromatin-binding domains are, to our knowledge, WDR5 and KAT8: WDR5 contains WD40 motifs (40) and KAT8 contains a chromo barrel motif with potential binding to methylated residues (41). We think that it is unlikely that these proteins are the components that compensate for the loss of PHF20 and PH20L1 in targeting the complex to chromatin; the WD40 binding profile (42) is different than that of PHF20 and PHF20L1, and the chromo barrel domain of KAT8 was shown to be required for its catalytic activity but not for its binding to chromatin in the context of MSL (43). We therefore favor scenario number 2; PHF20 and PHF20L1 have no enzymatic activity and strongly bind to methyl-lysine residues. Further supporting this scenario is our finding that PHF20 and PHF20L1 share the same DNA-binding motif described to be the binding motif of KANSL3 ((15) and Fig. S8). As for identifying potential compensatory mechanisms that target KANSL3 in the absence of PHF20 and/or PHF20L1, further studies will be needed. A good candidate is 53BP1, which has Tudor domains with a binding profile very similar to that of PHF20 (24) and has been shown to complex with KAT8 (44).

In this study, we focus on the functions of PHF20 and PHF20L1 in the context of NSL. It is likely that these proteins have functions outside this transcriptional complex. Our MS data show a few more proteins that are bound by PHF20 and PHF20L1 (Table S1) and are not part of NSL. For PHF20, these include RAS-responsive element binding protein 1, a RAS transcriptional effector shown to be a key partner of transforming growth factor-β-activated transcription factors (45). It will be interesting to test if PHF20 regulates transcription of RAS target genes in conditions where this pathway is activated.

Among other proteins with which PHF20L1 binds outside the NSL complex is CHD1, a chromatin remodeling factor that alters nucleosome positioning and facilitates DNA transcription and replication (46). The *CHD1* gene is considered a tumor suppressor in prostate cancer and contributes to transcriptional reprogramming by altering androgen receptor binding at lineage-specific enhancers (47). Further studies will define the role of PHF20L1 in the transcriptional regulation of androgen receptor signaling through its potential interactions with CHD1.

## Experimental procedures

### Cell lines and culture conditions

#### Human cancer cell line

Human osteosarcoma cell line U2OS was maintained in McCoy's 5A medium (catalog no.: 10-050-CV; Corning) supplemented with 10% Foundation fetal bovine serum (FBS) (catalog no.: 900-108; GeminiBio) or tetracycline-negative FBS (catalog no.: 100-800; GeminiBio) and 1% penicillin–streptomycin (P/S) (catalog no.: 113-98-43810-74-0; HyClone)

#### Primary MEF derivation

Embryos from PHF20 wildtype and PHF20 knockout mice were isolated between E12.5 and E18.5. After the heads, tails, limbs, and most of the internal organs were removed, the embryos were minced and incubated at 37 °C with 0.05% trypsin/EDTA (catalog no.: MT 25052CI; Corning) for 10 min and then seeded into 15 cm cell culture dishes in 20 ml of complete MEF media (Dulbecco's modified Eagle's medium [catalog no.: 10017CV; Corning] supplemented with 10% FBS, 1% P/S, and 0.1 mM β-mercaptoethanol). The cells were split at 1:2 to 1:3 ratios when freshly confluent and then frozen or expanded for further studies.

#### Generation of immortalized MEFs

BOSC23 cells were transfected with pBABE-puro SV40 LT (plasmid no.: 13970; Addgene) and pCL-Eco helper plasmid using XtremeGene9 DNA transfection reagent (catalog no.: 06365787001; Roche). After 48 h, the viral supernatant was filtered with a 0.45 micron filter and used to infect MEFs. MEFs were plated on a 6-well plate or 10 cm plate and grown for at least 24 h. On the day of infection, viral media containing 50% complete MEF media and 50% viral media with 4 μg/ml polybrene infection/transfection reagent (catalog no.: TR-1003-G; Millipore) were added, and cells were incubated for 48 h in viral media before letting recover from infection in fresh complete MEF media. Cells were selected with 1 μg/ml puromycin (catalog no.: P9620; Sigma) for at least 48 h before being used for experiments.

### Plasmid generation

#### Overexpression constructs

The pLOC lentiviral expression vectors were gifted by Dr Shawn Bratton. As described previously (48), FLAG tag was cloned into the BamHI–NheI sites of pLOC to generate an N-terminal FLAG-tag (pLOC-NFlag) vector or into the NheI/AscI sites of pLOC to generate a C-terminal FLAG tag (pLOC-CFlag). 3xHA tag was amplified by PCR, subcloned, and replaced FLAG tag in the pLOC vectors to generate an N-terminal or C-terminal 3xHA-tag vector (pLOC-N-3xHA and pLOC-C-3xHA, respectively).

FL *PHF20* and *PHF20L1* complementary DNA was constructed in the pEGFP-C1 vector by Biomatik and was a gift from Dr Mark T. Bedford. FL *PHF20* and *PHF20L1* complementary DNA was amplified by PCR and subcloned into the NheI/AscI sites of pLOC-NFlag and into the BamHI/NheI sites of pLOC-CFlag. FL (amino acids 1–1012 for PHF20, amino acids 1–1017 for PHF20L1), ΔC-term (amino acids 1–710 for PHF20, amino acids 1–750 for PHF20L1), and Tudors (amino acids 1–150 for PHF20 and PHF20L1) versions of *PHF20* and *PHF20L1* were then amplified by PCR and subcloned into the NheI/AscI sites of pLOC-N-3xHA. To generate ΔM versions of PHF20 and PHF20L1, two DNA fragments corresponding to amino acids 1 to 150 segment (for both PHF20 and PHF20L1) and to amino acids 650 to 1012 segment (for PHF20) or amino acids 650 to 1017 segment (for PHF20L1) were amplified by PCR and assembled into the pLOC-N-3xHA vectors using NEBuilder HiFi DNA Assembly Master Mix (catalog no.: E2621S; NEB). Point mutations in Tudor domains and PHD domain of *PHF20* and *PHF20L1* were subsequently introduced by site-directed mutagenesis using PCR with high-fidelity Phusion polymerase (catalog no.: F-531S; Thermo Fisher Scientific). A nonmutated DNA template was then digested with DpnI restriction enzymes. Mutated PCR products were then transformed into competent cells.

#### shRNA constructs

shRNAs in pGIPz vector backbones were synthesized by UT MD Anderson Cancer Center Functional Genomics Core. To generate inducible shRNA plasmids, shRNAs were subcloned from pGIPz to pTRIPz vector backbones using MluI and XhoI enzymes. pLKO.1 hygro was purchased from Addgene (plasmid no.: 24150; Addgene). shRNA sequences targeting 3′-UTR regions of PHF20 and PHF20L1 were taken from Sigma MISSION shRNA design (Sigma–Aldrich). Chosen shRNA sequences were subcloned into pLKO.1 hygro vector backbone using AgeI and EcoRI restriction sites. shRNA sequences used in this study are listed in Table S2.

### Generation of stable overexpression and KD cell lines

#### Generation of lentiviral media

*Chlorocebus aethiops* (Green monkey) COS1 cells were maintained in Dulbecco's modified Eagle's medium (catalog no.: 10-017-CV; Corning) supplemented with 10% FBS and 1% P/S. For transfection, 0.4 × 10^6^ COS1 cells were plated per well in a 6-well plate. About 24 h later, when cells are about 80% confluency, they were transfected with 1 μg pPAX.2, 1 μg pMD2G, and 1 μg experimental plasmid DNA with the *Trans*IT-2020 transfection reagent (catalog no.: MIR5400; Mirus) in Opti-MEM-I (catalog no.: 31985-070; Gibco), according to the manufacturer's instructions. Cells were incubated for 18 to 22 h before fresh media supplemented with 30% FBS and 1% P/S were added. Cells were incubated in high FBS media for 48 h, and viral media were collected. Viral media were filtered with a 0.45 micron filter and used to infect U2OS cells.

#### Lentiviral infection

U2OS cells were plated on a 6-well plate or 10 cm plate and grown for at least 24 h. On the day of infection, viral media containing 50% complete media and 50% viral media with 10 μg/ml Polybrene Infection/Transfection reagent (catalog no.: TR-1003-G; Millipore) were added, and cells were incubated for 48 h in viral media. Cells were harvested and replated in complete media with 15 μg/ml blasticidin (catalog no.: ant-bl-5b; InvivoGen), 1 μg/ml puromycin (catalog no.: P9620; Sigma–Aldrich), or 50 μg/ml hygromycin B (catalog no.: 10687-010; Invitrogen). Cells were selected until all cells in the noninfected plate were killed by antibiotics or by sorting for GFP-positive cell populations by BD FACSAria II Cell Sorter (BD). Cells were then harvested or used for experiments.

#### Dox induction

Cells that were subjected to Dox induction were maintained in media containing tetracycline-negative FBS. Cells were then treated with 2 μg/ml Dox (catalog no.: D3072; Sigma–Aldrich) for 5 days, with Dox being replenished daily. Cells induced by Dox were sorted as red fluorescent protein–positive cell populations by BD FACSAria II Cell Sorter. Cells were then used for experiments or alternatively maintained in Dox for additional 9 days before harvested for experiments.

#### IP

Cells were lysed in mild lysis buffer (MLB, 50 mM Tris–HCl, pH 7.5, 150 mM NaCl, 0.1% NP-40, 5 mM EDTA, 5 mM EGTA, and 15 mM MgCl_2_) supplemented with HALT protease inhibitor cocktail (PIs; catalog no.: 78438; Thermo Fisher Scientific) and phosphatase inhibitors (PhIs; 1 mM sodium orthovanadate, 1 mM sodium molybdate, 4 mM sodium tartrate, 1 mM sodium fluoride, 2 mM imidazole, 2 mM β-glycerophosphate, and 1 mM sodium pyrophosphate). Protein concentration of lysates was measured by bicinchoninic acid (catalog no.: 23225; Pierce). About 500 μg to 1 mg of protein was precleared with hydrated Protein A Sepharose beads (catalog no.: 17-0780-01; GE Healthcare) for 1.5 h at 4 °C. Meanwhile, FLAG M2 beads (catalog no.: A2220; Sigma–Aldrich) or Pierce Anti-HA Magnetic Beads (catalog no.: 88836; Thermo Fisher Scientific) were washed three times with MLB. Precleared lysates were then incubated with washed FLAG M2 beads or Pierce Anti-HA Magnetic Beads overnight at 4 °C. Beads with bound protein complexes were washed three times with MLB. IP’ed products were sent out for MS or resolved by SDS-PAGE and subjected to WB analysis. For MS analysis, the last wash contained 250 mM NaCl, and bound protein complexes were eluted from the beads by incubating the beads in elution buffer (5% SDS, 50 mM Tris, pH 7.5) at 37 °C for 20 min. For WB analysis, bound protein complexes were eluted from the beads by boiling the beads in 2× Laemmli loading buffer (catalog no.: 1610737; Bio-Rad) with 5% β-mercaptoethanol for 5 min.

#### MS

The samples were prepared as described previously (49). Brieﬂy, the agarose bead-bound proteins were washed several times with 50 mM triethylammonium bicarbonate (TEAB) at pH 7.1, before being solubilized with 40 ml of 5% SDS, 50 mM TEAB, pH 7.55 followed by a room temperature incubation for 30 min. The supernatant containing the proteins of interest was then transferred to a new tube, reduced by making the solution 10 mM Tris(2-carboxyethyl)phosphine (catalog no.: 77720; Thermo Fisher Scientific), and further incubated at 65 °C for 10 min. The sample was then cooled to room temperature, and 3.75 ml of 1 M iodoacetamide acid was added and allowed to react for 20 min in the dark after which 0.5 ml of 2 M DTT was added to quench the reaction. Then, 5 ml of 12% phosphoric acid was then added to the 50 ml protein solution followed by 350 ml of binding buffer (90% methanol, 100 mM TEAB final; pH 7.1). The resulting solution was administered to an S-Trap spin column (Protifi) and passed through the column using a bench-top centrifuge (30 s spin at 4000*g*). The spin column was then washed three times with 400 ml of binding buffer and centrifuged (1200 rpm, 1 min). Trypsin (catalog no.: V5280; Promega) was then added to the protein mixture in a ratio of 1:25 in 50 mM TEAB, pH = 8, and incubated at 37 °C for 4 h. Peptides were eluted with 80 μl of 50 mM TEAB, followed by 80 ml of 0.2% formic acid, and finally 80 ml of 50% acetonitrile (ACN) and 0.2% formic acid. The combined peptide solution was then dried in a speed vacuum (room temperature, 1.5 h) and resuspended in 2% ACN, 0.1% formic acid, 97.9% water, and aliquoted into an autosampler vial

#### NanoLC–MS/MS analysis

Peptide mixtures were analyzed by nanoLC–MS/MS using a nano-LC chromatography system (UltiMate 3000 RSLCnano; Dionex, Thermo Fisher Scientific). The nanoLC–MS/MS system was coupled online to a Thermo Orbitrap Fusion mass spectrometer (Thermo Fisher Scientific) through a nanospray ion source (Thermo Fisher Scientific). A trap-and-elute method was used to desalt and concentrate the sample, while preserving the analytical column. The trap column (Thermo Fisher Scientific) was a C18 PepMap100 (300 μm × 5 mm, 5 μm particle size), whereas the analytical column was an Acclaim PepMap 100 (75 mm × 25 cm) (Thermo Fisher Scientific). After equilibrating the column in 98% solvent A (0.1% formic acid in water) and 2% solvent B (0.1% formic acid in ACN), the samples (2 ml in solvent A) were injected onto the trap column and subsequently eluted (400 nl/min) by gradient elution onto the C18 column as follows: isocratic at 2% B, 0 to 5 min; 2% to 32% B, 5 to 39 min; 32% to 70% B, 39 to 49 min; 70% to 90% B, 49 to 50 min; isocratic at 90% B, 50 to 54 min; 90% to 2%, 54 to 55 min; and isocratic at 2% B, until the 65 min mark. All LC–MS/MS data were acquired using XCalibur, version 2.1.0 (Thermo Fisher Scientific) in positive ion mode using a top speed data-dependent acquisition method with a 3 s cycle time. The survey scans (*m/z* 350–1500) were acquired in the Orbitrap at 120,000 resolution (at *m/z* = 400) in profile mode, with a maximum injection time of 100 ms and an automatic gain control target of 400,000 ions. The S-lens radiofrequency level was set to 60. Isolation was performed in the quadrupole with a 1.6 Da isolation window, and collision-induced dissociation MS/MS acquisition was performed in profile mode using rapid scan rate with detection in the ion trap using the following settings: parent threshold = 5000; collision energy = 32%; maximum injection time = 56 ms; automatic gain control target = 500,000 ions. Monoisotopic precursor selection and charge state filtering were on, with charge states 2 to 6 included. Dynamic exclusion was used to remove selected precursor ions, with a ± 10 ppm mass tolerance, for 15 s after acquisition of one MS/MS spectrum

#### Database searching

Tandem mass spectra were extracted, and charge state was deconvoluted using Proteome Discoverer (version 2.4.035; Thermo Fisher Scientific). Deisotoping was not performed. All MS/MS spectra were searched against a UniProt Human database (version 06-2019; 20,369 entries) using Sequest. Searches were performed with a parent ion tolerance of 5 ppm and a fragment ion tolerance of 0.60 Da. Trypsin was specified as the enzyme, allowing for two missed cleavages. Fixed modiﬁcation of carbamidomethyl (C) and variable modifications of oxidation (M) and deamidation were specified in Sequest. Search results were imported into Scaffold (Proteome Software) and searched with X!Tandem Alanine (2017.2.4) using the same conditions as described previously. False discovery rate was calculated using the decoy method. Protein identifications were accepted if they could be established at greater than 95.0% probability and contained at least two identified peptides.

### Subcellular protein fractionation

Cells were swollen in hypotonic cytoplasm extraction buffer (10 mM Hepes, pH 7.8, 10 mM KCl, 1.5 mM MgCl_2_, 10% glycerol, and 340 mM sucrose) supplemented with PIs and PhIs for 15 min on ice. After 15 min of swelling, NP-40 and Triton X-100 were added to the final concentration of 0.25% and 0.1%, respectively. The cell lysates were then vortexed for 5 s after detergents were added. Cytoplasmic fractions were separated from nuclei pellets by centrifugation at 13,000*g* for 5 min at 4 °C. Nuclei pellets were washed once with hypotonic cytoplasm extraction buffer supplemented with PIs, PhIs, 0.25% NP-40, and 0.1% Triton X-100. Nuclei pellets were then lysed in hypotonic nuclear lysis buffer (3 mM EDTA, 0.2 mM EGTA, 0.5% NP-40, and 500 mM NaCl), supplemented with PIs and PhIs, and incubated on ice for 30 min with a 15 s vortex every 10 min. At the end of the 30 min incubation, the nuclei lysates were dounced twice with plastic pastels. The soluble nuclear fractions were collected as supernatants after a centrifugation at 1700*g* for 5 min at 4 °C. The chromatin pellets were washed once with hypotonic nuclear lysis buffer supplemented with PIs and PhIs. The chromatin pellets were then resuspended in chromatin-bound protein extraction buffer (10 mM Hepes, pH 7.6–7.9, 500 mM NaCl, 10 mM KCl, 10% glycerol, 2 mM MgCl_2_, 5 mM CaCl_2_, and 1 mM EDTA) supplemented with PIs and PhIs. 5 U of micrococcal nuclease (catalog no.: 88216; Thermo Fisher Scientific) were added to the chromatin lysates, and the chromatin lysates were incubated for 1 h at 37 °C. EGTA was added to the final concentration of 1 mM to stop the micrococcal nuclease reactions at the end of the incubation. All protein fractions were centrifuged at 20,000*g* for 10 min at 4 °C for final protein collection. Protein fractions were resolved by SDS-PAGE and subjected to WB analysis.

### WB

For whole-cell lysate WBs, cells were lysed in IPH buffer (50 mM Tris–HCl, pH 7.5, 150 mM NaCl, 5 mM EDTA, 10% glycerol, 0.5% NP-40, and 0.01% SDS) with supplemented with PIs and PhIs and then subjected to sonication using pulsations (30 s on, 30 s off) for 3 min at 4 °C. Protein concentrations were determined by bicinchoninic acid (catalog no.: 23225; Pierce). About 30 to 50 μg protein was resolved by SDS-PAGE and wet transferred to nitrocellulose for blotting. Immunoblots were blocked with 3% milk in Tris-buffered saline (TBS) solution (3% milk/TBS) for 1 h at room temperature and then incubated with primary antibody overnight at 4 °C. All antibodies were prepared in 3% milk/TBS. Following three washes in TBS buffer containing 0.1% Tween-20, the blots were further incubated with IRDye 800CW Goat anti-Rabbit immunoglobulin G (IgG) Secondary Antibody (1:15,000 dilution; catalog no.: 926-32211; LI-COR) or IRDye 680LT Goat anti-Mouse IgG Secondary Antibody (1:20,000 dilution; catalog no.: 926-68020; LI-COR) at room temperature for 2 h. Fluorescence signals were visualized using LI-COR Odyssey Imaging System (LI-COR). All experiments were performed at least in duplicates to confirm the results. Primary antibodies with the respective dilution factors are as following: HA tag (C29F4) (1:1000 dilution; catalog no.: 3724), PHF20 (D96F6) XP (1:1000 dilution; catalog no.: 3934), WDR5 (D9E1I) (1:1000 dilution; catalog no.: 13105), KAT8/MYST1 (D5T3R) (1:1000 dilution; catalog no.: 46862) (from Cell Signaling Technology), PHF20L1 (1:500 dilution; catalog no.: HPA028417), KANSL3 (1:1000 dilution; catalog no.: HPA035018), β-actin (1:1000 dilution; catalog no.: A1978) (from Sigma–Aldrich), lamin B1 (1:5000 dilution; catalog no.: ab133741), histone H2B (1:5000 dilution; catalog no.: ab52484) (from Abcam), and GAPDH (1:1000 dilution; catalog no.: GT239) (from Genentech).

### ChIP–qPCR

Cells were grown to 60 to 80% confluence and crosslinked with 1% formaldehyde (catalog no.: F8775; Sigma–Aldrich) for 10 min. The crosslinking reaction was quenched by 0.125 M glycine. The fixed cells were then washed twice with ice-cold PBS and scraped out of the plate in ice-cold PBS supplemented with PIs and PhIs. Nuclei were isolated from formaldehyde-crosslinked cells in cell lysis buffer (5 mM Pipes, pH 8.0, 85 mM KCl, 1% NP-40 supplemented with PIs and PhIs). The nuclei were lysed in nuclei lysis buffer (50 mM Tris–HCl, pH 8.0, 10 mM EDTA, 1% SDS supplemented with PIs and PhIs) and subjected to sonication with a Bioruptor Sonicator (Diagenode) to obtain DNA fragments ranging 200 to 600 bp. Chromatin lysates were then precleared and incubated with either sheep IgG isotype control (catalog no.: 31243; Thermo Fisher Scientific) or respective antibodies in lysis buffer (150 mM NaCl, 25 mM Tris–HCl, pH 7.5, 5 mM EDTA, 1% Triton X-100, 0.1% SDS, 0.5% sodium deoxycholate, supplemented with PIs and PhIs) overnight at 4 °C. The next day, Dynabeads Protein A (catalog no.: 10002D; Invitrogen) were added into the reactions and incubated for 2 h at 4 °C. The beads were then washed once with each of the following buffer: radioimmunoprecipitation assay (50 mM Tris–HCl, pH 8.0, 150 mM NaCl, 0.1% SDS, 0.5% sodium deoxycholate, 1% NP-40, 1 mM EDTA, supplemented with PIs and PhIs), high salt buffer (50 mM Tris–HCl, pH 8.0, 500 mM NaCl, 0.1% SDS, 0.5% sodium deoxycholate, 1% NP-40, 1 mM EDTA, supplemented with PIs and PhIs), LiCl Wash (50 mM Tris–HCl, pH 8.0, 1 mM EDTA, 250 mM LiCl, 1% NP-40, 0.5% sodium deoxycholate, supplemented with PIs and PhIs), 10 mM Tris–HCl, pH 8.0, 1 mM EDTA (supplemented with PIs and PhIs), and 10 mM Tris–HCl, pH 8.0, 1 mM EDTA without PIs and PhIs. The samples were then subjected to RNAse A treatment for 1 h at 37 °C, proteinase K treatment for 4 h at 37 °C, and reverse crosslinking overnight at 65 °C, followed by DNA extraction using phenol/chloroform. Per ChIP, 25 to 100 μg of chromatin was used. Per ChIP, 5 μg of KANSL3 antibody (catalog no.: HPA035018; Sigma–Aldrich), 1 μg of HA-tag antibody (catalog no.: 3724; Cell Signaling Technology), 3 μg of H4K5ac antibody (catalog no.: ab51997), 3 μg of H4K8ac antibody (catalog no.: ab45166), or 3 μg of histone H3 antibody (catalog no.: ab1791) (from Abcam) were used. The corresponding amount of sheep IgG isotype control was used as a negative control. For qPCR analysis, real-time PCR was performed with the iTaq SYBRgreen Supermix (catalog no.: 1725121; Bio-Rad) on an ABI 7900HT Fast Real-Time PCR system, according to the manufacturer's protocol. Different biological replicates were performed and analyzed. Samples were normalized to percent of input. ChIP–qPCR primers are listed in Table S2. HA-ChIPs and library preparation for sequencing PHF20-bound and PHF20L-bound DNA (ChIP-Seq) were performed at MD Anderson Epigenomics Profiling Core as described earlier (50). Libraries for ChIP DNA were prepared using the NEBNext Ultra II DNA Library Prep Kit for Illumina (catalog no.: E7645S; NEB). ChIP DNA and the corresponding input DNA libraries were sequenced on Illumina NextSeq 500 instrument to obtain 30 to 40 million 50 bp single reads per sample.

### RNA extraction, RT–qPCR

Cells were spun down and washed once with cold PBS. Then RNA was isolated with a QIAGEN RNeasy kit (catalog no.: 74104; QIAGEN) according to the manufacturer's protocol. About 1 μg of total RNA was treated with amplification-grade DNase I (catalog no.: 79254; QIAGEN) according to the manufacturer's protocol and reverse transcribed with the SuperScript III Supermix system (catalog no.: 11752-250; Invitrogen), according to the manufacturer's protocol. mRNA levels were analyzed *via* qPCR analysis using the iTaq SYBR Green Supermix (catalog no.: 1725121; Bio-Rad), according to the manufacturer's protocol. Three biological replicates were analyzed, with two to three technical replicates per plate. Reactions were performed on an ABI 7900HT Fast Real-Time PCR system. Samples were normalized to transcripts encoding the *RPLP0* gene (for human cells) and *Rplp0* (for MEFs). qPCR primers are listed in Table S2.

### ChIP-Seq

HA ChIPs for ChIP-Seq were performed at the MD Anderson Epigenomics Profiling Core in a high-throughput format. ChIP-Seq libraries were made from ChIP DNA using the NEBNext Ultra II DNA library prep kit (NEB). The ChIP-Seq libraries and the corresponding input libraries were sequenced using 50 bases single-read protocol on Illumina NextSeq 500 and HiSeq 3000 instrument. Three biological replicates per protein were sequenced, generating 36 to 89 million reads per sample

#### Mapping

Sequenced DNA reads were mapped to the human genome (hg38) using Bowtie (51) (version 1.1.2), and only the reads that were mapped to unique position were retained. About 93 to 97% reads were mapped to the human genome, with 81 to 84% uniquely mapped. To avoid PCR bias, for multiple reads that were mapped to the same genomic position, only one copy was retained for further analysis. About 23 to 66 million reads were finally used in peak calling and downstream analyses.

#### Peak calling

The peaks in each ChIP sample were detected by MACS2 (52) (version 2.2.7.1) by comparing to the corresponding input sample. The --extsize was set as 200 bp. The peaks that overlapped ENCODE blacklist regions (53) were removed. One of PHF20 replicates failed in peak calling with few peaks called, thus this replicate was removed from further analysis. The remaining replicates of PHF20 or PHF20L1 also varied in terms of signal intensity. The peaks were called in the replicate of PHF20 or PHF20L1 with best signal at *p* value ≤0.01 and in the other replicate(s) at *p* value ≤0.05. The peaks that were called in the best replicate and overlapped the peaks called in the other replicate(s) were identified as the final list of PHF20-binding or PHF20L1-binding sites. Totally, 4147 peaks and 723 peaks were called for PHF20 and PHF20L1, respectively. About 91.7% of PHF20 peaks and 92.5% of PHF20L1 peaks were found in the promoter region (defined as −1 k to +0.5 k of TSS)

#### Genomic distribution of peaks

Each peak was assigned to the gene that has the closest TSS to it. Then the peak was classified by its location to the gene: upstream (−5 kb to −1 kb from TSS), promoter (−1 kb to +0.5 kb from TSS), exon, intron, transcription end site (TES) (−0.5 kb to +1 kb from TES), downstream (+1 kb to +5 kb from TES), and intergenic. Genes from GENCODE, Release 35 (54) were used for the annotation

#### Signal landscape

Each read was extended by 200 bp to its 3′ end. The pileup of reads on each genomic position was normalized to a total of 10 M mapped reads, averaged over every 10 bp window, and displayed in UCSC genome browser (55)

#### Processing of public KANSL3 ChIP-Seq data

The list of 373 KANSL3 peaks was downloaded from https://pubmed.ncbi.nlm.nih.gov/33657400/.

#### Venn diagram of peak sets

The peaks from the two or three peak sets shown in the Venn diagram were merged, allowing at least 1 bp overlap. Then the numbers of merged peaks that overlapped different intersections of the peak sets were presented in the Venn diagram.

#### Aggregated signal profile over TSS

About −2.5 kb to +2.5 kb of a TSS were subdivided into 10 bp bins. For each ChIP or input sample, the pileup of reads after the normalization to a total of 10 M mapped reads was averaged for each bin. Then the pileup of reads was averaged over all TSSs and plotted.

#### Motif analysis

Motif analysis was done by MEME-ChIP program (56) from MEME Suite (57) (version 5.3.3). The sequences of −500 bp to +500 bp around peak summits were taken as input.

### RNA-Seq

For gene expression data, the raw fastq files of two replicates of U2OS RNA-Seq were downloaded (58) by TopHat (version 2.0.10). The number of reads mapped to each known gene in GENCODE Release 35 was enumerated using htseq-count from HTSeq package (version 0.6.0). Genes shorter than 200 bp, coding for rRNAs, and on chromosome Y were removed. The number of fragments per kilobase per million fragments value for each gene was calculated and averaged over the two replicates.

### Statistical analysis

Multiple independent biological experiments were performed to assess the reproducibility of experimental findings. For analysis of gene expression, the measured cycle threshold experimental values for each transcript were compiled and normalized to the *RPLP0* or *Rplp0* reference gene. Normalized expression levels were calculated using the delta delta cycle threshold method described previously (59). Values from these calculations were transferred into the statistical analysis program GraphPad (GraphPad Software, Inc). One-way ANOVA (multiple comparison function) or unpaired *t* test was run to assay differences between control and experimental samples. For quantitative analysis of protein enrichment at the specific TSSs, ChIP samples were normalized to 1% input, and data were analyzed using the formula previously described (60). The cumulative mean from each of the independent experiments was calculated, and the standard deviation of the mean was derived. Two-way ANOVA (multiple comparison function) was run to assay differences between control and experimental samples. Data were represented as mean ± SD. For samples with *p* values <0.05, we have marked statistically significant differences with asterisks and denoted the *p* value ranges in figure legends. *p* Values were represented as ∗*p* < 0.05, ∗∗*p* < 0.01, ∗∗∗*p* < 0.001, and ∗∗∗∗*p* < 0.0001.

## Data availability

All raw and normalized RNA-Seq and ChIP-Seq data that support the findings of this study have been deposited in the GEO SuperSeries under accession number GSE188601. The MS proteomics data have been deposited to the ProteomeXchange Consortium *via* the PRIDE (61) partner repository with the dataset identifier PXD029258 and 10.6019/PXD029258.

## Supporting information

This article contains supporting information.

## Conflict of interest

The authors declare that they have no conflicts of interest with the contents of this article.

## References

[1] Maniatis T., Reed R. (2002). An extensive network of coupling among gene expression machines. Nature.

[2] Akhtar A., Becker P.B. (2000). Activation of transcription through histone H4 acetylation by MOF, an acetyltransferase essential for dosage compensation in Drosophila. Mol. Cell.

[3] Hilfiker A., Hilfiker-Kleiner D., Pannuti A., Lucchesi J.C. (1997). mof, a putative acetyl transferase gene related to the Tip60 and MOZ human genes and to the SAS genes of yeast, is required for dosage compensation in Drosophila. EMBO J..

[4] Belote J.M., Lucchesi J.C. (1980). Male-specific lethal mutations of Drosophila melanogaster. Genetics.

[5] Tanaka A., Fukunaga A., Oishi K. (1976). Studies on the sex-specific lethals of Drosophila melanogaster. II. Further studies on a male-specific lethal gene, maleless. Genetics.

[6] Bone J.R., Kuroda M.I. (1996). Dosage compensation regulatory proteins and the evolution of sex chromosomes in Drosophila. Genetics.

[7] Hilfiker A., Yang Y., Hayes D.H., Beard C.A., Manning J.E., Lucchesi J.C. (1994). Dosage compensation in Drosophila: The X-chromosomal binding of MSL-1 and MLE is dependent on Sxl activity. EMBO J..

[8] Sheikh B.N., Guhathakurta S., Akhtar A. (2019). The non-specific lethal (NSL) complex at the crossroads of transcriptional control and cellular homeostasis. EMBO Rep..

[9] Feller C., Prestel M., Hartmann H., Straub T., Soding J., Becker P.B. (2012). The MOF-containing NSL complex associates globally with housekeeping genes, but activates only a defined subset. Nucleic Acids Res..

[10] Lam K.C., Muhlpfordt F., Vaquerizas J.M., Raja S.J., Holz H., Luscombe N.M., Manke T., Akhtar A. (2012). The NSL complex regulates housekeeping genes in Drosophila. PLoS Genet..

[11] Raja S.J., Charapitsa I., Conrad T., Vaquerizas J.M., Gebhardt P., Holz H., Kadlec J., Fraterman S., Luscombe N.M., Akhtar A. (2010). The nonspecific lethal complex is a transcriptional regulator in Drosophila. Mol. Cell.

[12] Gaub A., Sheikh B.N., Basilicata M.F., Vincent M., Nizon M., Colson C., Bird M.J., Bradner J.E., Thevenon J., Boutros M., Akhtar A. (2020). Evolutionary conserved NSL complex/BRD4 axis controls transcription activation via histone acetylation. Nat. Commun..

[13] Cai Y., Jin J., Swanson S.K., Cole M.D., Choi S.H., Florens L., Washburn M.P., Conaway J.W., Conaway R.C. (2010). Subunit composition and substrate specificity of a MOF-containing histone acetyltransferase distinct from the male-specific lethal (MSL) complex. J. Biol. Chem..

[14] Zhao X., Su J., Wang F., Liu D., Ding J., Yang Y., Conaway J.W., Conaway R.C., Cao L., Wu D., Wu M., Cai Y., Jin J. (2013). Crosstalk between NSL histone acetyltransferase and MLL/SET complexes: NSL complex functions in promoting histone H3K4 di-methylation activity by MLL/SET complexes. PLoS Genet..

[15] Radzisheuskaya A., Shliaha P.V., Grinev V.V., Shlyueva D., Damhofer H., Koche R., Gorshkov V., Kovalchuk S., Zhan Y., Rodriguez K.L., Johnstone A.L., Keogh M.C., Hendrickson R.C., Jensen O.N., Helin K. (2021). Complex-dependent histone acetyltransferase activity of KAT8 determines its role in transcription and cellular homeostasis. Mol. Cell.

[16] Mendjan S., Taipale M., Kind J., Holz H., Gebhardt P., Schelder M., Vermeulen M., Buscaino A., Duncan K., Mueller J., Wilm M., Stunnenberg H.G., Saumweber H., Akhtar A. (2006). Nuclear pore components are involved in the transcriptional regulation of dosage compensation in Drosophila. Mol. Cell.

[17] Sharma G.G., So S., Gupta A., Kumar R., Cayrou C., Avvakumov N., Bhadra U., Pandita R.K., Porteus M.H., Chen D.J., Cote J., Pandita T.K. (2010). MOF and histone H4 acetylation at lysine 16 are critical for DNA damage response and double-strand break repair. Mol. Cell. Biol..

[18] Adams-Cioaba M.A., Li Z., Tempel W., Guo Y., Bian C., Li Y., Lam R., Min J. (2012). Crystal structures of the Tudor domains of human PHF20 reveal novel structural variations on the Royal Family of proteins. FEBS Lett..

[19] Cui G., Park S., Badeaux A.I., Kim D., Lee J., Thompson J.R., Yan F., Kaneko S., Yuan Z., Botuyan M.V., Bedford M.T., Cheng J.Q., Mer G. (2012). PHF20 is an effector protein of p53 double lysine methylation that stabilizes and activates p53. Nat. Struct. Mol. Biol..

[20] Klein B.J., Wang X., Cui G., Yuan C., Botuyan M.V., Lin K., Lu Y., Wang X., Zhao Y., Bruns C.J., Mer G., Shi X., Kutateladze T.G. (2016). PHF20 readers link methylation of histone H3K4 and p53 with H4K16 acetylation. Cell Rep..

[21] Park S., Kim D., Dan H.C., Chen H., Testa J.R., Cheng J.Q. (2012). Identification of Akt interaction protein PHF20/TZP that transcriptionally regulates p53. J. Biol. Chem..

[22] Zhang T., Park K.A., Li Y., Byun H.S., Jeon J., Lee Y., Hong J.H., Kim J.M., Huang S.M., Choi S.W., Kim S.H., Sohn K.C., Ro H., Lee J.H., Lu T. (2013). PHF20 regulates NF-kappaB signalling by disrupting recruitment of PP2A to p65. Nat. Commun..

[23] Zhang X., Peng D., Xi Y., Yuan C., Sagum C.A., Klein B.J., Tanaka K., Wen H., Kutateladze T.G., Li W., Bedford M.T., Shi X. (2016). G9a-mediated methylation of ERalpha links the PHF20/MOF histone acetyltransferase complex to hormonal gene expression. Nat. Commun..

[24] Badeaux A.I., Yang Y., Cardenas K., Vemulapalli V., Chen K., Kusewitt D., Richie E., Li W., Bedford M.T. (2012). Loss of the methyl lysine effector protein PHF20 impacts the expression of genes regulated by the lysine acetyltransferase MOF. J. Biol. Chem..

[25] Bankovic J., Stojsic J., Jovanovic D., Andjelkovic T., Milinkovic V., Ruzdijic S., Tanic N. (2010). Identification of genes associated with non-small-cell lung cancer promotion and progression. Lung Cancer.

[26] Fischer U., Struss A.K., Hemmer D., Pallasch C.P., Steudel W.I., Meese E. (2001). Glioma-expressed antigen 2 (GLEA2): A novel protein that can elicit immune responses in glioblastoma patients and some controls. Clin. Exp. Immunol..

[27] Heisel S.M., Ketter R., Keller A., Klein V., Pallasch C.P., Lenhof H.P., Meese E. (2008). Increased seroreactivity to glioma-expressed antigen 2 in brain tumor patients under radiation. PLoS One.

[28] Taniwaki M., Daigo Y., Ishikawa N., Takano A., Tsunoda T., Yasui W., Inai K., Kohno N., Nakamura Y. (2006). Gene expression profiles of small-cell lung cancers: Molecular signatures of lung cancer. Int. J. Oncol..

[29] Schulte I., Batty E.M., Pole J.C., Blood K.A., Mo S., Cooke S.L., Ng C., Howe K.L., Chin S.F., Brenton J.D., Caldas C., Howarth K.D., Edwards P.A. (2012). Structural analysis of the genome of breast cancer cell line ZR-75-30 identifies twelve expressed fusion genes. BMC Genomics.

[30] Hou Y., Liu W., Yi X., Yang Y., Su D., Huang W., Yu H., Teng X., Yang Y., Feng W., Zhang T., Gao J., Zhang K., Qiu R., Wang Y. (2020). PHF20L1 as a H3K27me2 reader coordinates with transcriptional repressors to promote breast tumorigenesis. Sci. Adv..

[31] Esteve P.O., Terragni J., Deepti K., Chin H.G., Dai N., Espejo A., Correa I.R., Bedford M.T., Pradhan S. (2014). Methyllysine reader plant homeodomain (PHD) finger protein 20-like 1 (PHF20L1) antagonizes DNA (cytosine-5) methyltransferase 1 (DNMT1) proteasomal degradation. J. Biol. Chem..

[32] Esteve P.O., Chin H.G., Benner J., Feehery G.R., Samaranayake M., Horwitz G.A., Jacobsen S.E., Pradhan S. (2009). Regulation of DNMT1 stability through SET7-mediated lysine methylation in mammalian cells. Proc. Natl. Acad. Sci. U. S. A..

[33] Zhang C., Leng F., Saxena L., Hoang N., Yu J., Alejo S., Lee L., Qi D., Lu F., Sun H., Zhang H. (2019). Proteolysis of methylated SOX2 protein is regulated by L3MBTL3 and CRL4(DCAF5) ubiquitin ligase. J. Biol. Chem..

[34] Carr S.M., Munro S., Sagum C.A., Fedorov O., Bedford M.T., La Thangue N.B. (2017). Tudor-domain protein PHF20L1 reads lysine methylated retinoblastoma tumour suppressor protein. Cell Death Differ..

[35] Wang Q., Yu M., Ma Y., Zhang X., Zhang H., Li S., Lan R., Lu F. (2018). PHF20L1 antagonizes SOX2 proteolysis triggered by the MLL1/WDR5 complexes. Lab. Invest..

[36] Zhang C., Hoang N., Leng F., Saxena L., Lee L., Alejo S., Qi D., Khal A., Sun H., Lu F., Zhang H. (2018). LSD1 demethylase and the methyl-binding protein PHF20L1 prevent SET7 methyltransferase-dependent proteolysis of the stem-cell protein SOX2. J. Biol. Chem..

[37] Natrajan R., Weigelt B., Mackay A., Geyer F.C., Grigoriadis A., Tan D.S., Jones C., Lord C.J., Vatcheva R., Rodriguez-Pinilla S.M., Palacios J., Ashworth A., Reis-Filho J.S. (2010). An integrative genomic and transcriptomic analysis reveals molecular pathways and networks regulated by copy number aberrations in basal-like, HER2 and luminal cancers. Breast Cancer Res. Treat..

[38] Wrzeszczynski K.O., Varadan V., Byrnes J., Lum E., Kamalakaran S., Levine D.A., Dimitrova N., Zhang M.Q., Lucito R. (2011). Identification of tumor suppressors and oncogenes from genomic and epigenetic features in ovarian cancer. PLoS One.

[39] Lv M., Gao J., Li M., Ma R., Li F., Liu Y., Liu M., Zhang J., Yao X., Wu J., Shi Y., Tang Y., Pan Y., Zhang Z., Ruan K. (2020). Conformational selection in ligand recognition by the first tudor domain of PHF20L1. J. Phys. Chem. Lett..

[40] Gori F., Divieti P., Demay M.B. (2001). Cloning and characterization of a novel WD-40 repeat protein that dramatically accelerates osteoblastic differentiation. J. Biol. Chem..

[41] Nielsen P.R., Nietlispach D., Buscaino A., Warner R.J., Akhtar A., Murzin A.G., Murzina N.V., Laue E.D. (2005). Structure of the chromo barrel domain from the MOF acetyltransferase. J. Biol. Chem..

[42] Schapira M., Tyers M., Torrent M., Arrowsmith C.H. (2017). WD40 repeat domain proteins: A novel target class?. Nat. Rev. Drug Discov..

[43] Conrad T., Cavalli F.M., Holz H., Hallacli E., Kind J., Ilik I., Vaquerizas J.M., Luscombe N.M., Akhtar A. (2012). The MOF chromobarrel domain controls genome-wide H4K16 acetylation and spreading of the MSL complex. Dev. Cell.

[44] Gupta A., Hunt C.R., Hegde M.L., Chakraborty S., Chakraborty S., Udayakumar D., Horikoshi N., Singh M., Ramnarain D.B., Hittelman W.N., Namjoshi S., Asaithamby A., Hazra T.K., Ludwig T., Pandita R.K. (2014). MOF phosphorylation by ATM regulates 53BP1-mediated double-strand break repair pathway choice. Cell Rep..

[45] Su J., Morgani S.M., David C.J., Wang Q., Er E.E., Huang Y.H., Basnet H., Zou Y., Shu W., Soni R.K., Hendrickson R.C., Hadjantonakis A.K., Massague J. (2020). TGF-beta orchestrates fibrogenic and developmental EMTs via the RAS effector RREB1. Nature.

[46] Farnung L., Vos S.M., Wigge C., Cramer P. (2017). Nucleosome-Chd1 structure and implications for chromatin remodelling. Nature.

[47] Augello M.A., Liu D., Deonarine L.D., Robinson B.D., Huang D., Stelloo S., Blattner M., Doane A.S., Wong E.W.P., Chen Y., Rubin M.A., Beltran H., Elemento O., Bergman A.M., Zwart W. (2019). CHD1 loss alters AR binding at lineage-specific enhancers and modulates distinct transcriptional programs to drive prostate tumorigenesis. Cancer Cell.

[48] Wible D.J., Chao H.P., Tang D.G., Bratton S.B. (2019). ATG5 cancer mutations and alternative mRNA splicing reveal a conjugation switch that regulates ATG12-ATG5-ATG16L1 complex assembly and autophagy. Cell Discov..

[49] Li L., Huang K.L., Gao Y., Cui Y., Wang G., Elrod N.D., Li Y., Chen Y.E., Ji P., Peng F., Russell W.K., Wagner E.J., Li W. (2021). An atlas of alternative polyadenylation quantitative trait loci contributing to complex trait and disease heritability. Nat. Genet..

[50] Jain A.K., Xi Y., McCarthy R., Allton K., Akdemir K.C., Patel L.R., Aronow B., Lin C., Li W., Yang L., Barton M.C. (2016). LncPRESS1 is a p53-regulated LncRNA that safeguards pluripotency by disrupting SIRT6-mediated de-acetylation of histone H3K56. Mol. Cell.

[51] Langmead B., Trapnell C., Pop M., Salzberg S.L. (2009). Ultrafast and memory-efficient alignment of short DNA sequences to the human genome. Genome Biol..

[52] Zhang Y., Liu T., Meyer C.A., Eeckhoute J., Johnson D.S., Bernstein B.E., Nusbaum C., Myers R.M., Brown M., Li W., Liu X.S. (2008). Model-based analysis of ChIP-seq (MACS). Genome Biol..

[53] Amemiya H.M., Kundaje A., Boyle A.P. (2019). The ENCODE blacklist: Identification of problematic regions of the genome. Sci. Rep..

[54] Frankish A., Diekhans M., Ferreira A.M., Johnson R., Jungreis I., Loveland J., Mudge J.M., Sisu C., Wright J., Armstrong J., Barnes I., Berry A., Bignell A., Carbonell Sala S., Chrast J. (2019). GENCODE reference annotation for the human and mouse genomes. Nucleic Acids Res..

[55] Kent W.J., Sugnet C.W., Furey T.S., Roskin K.M., Pringle T.H., Zahler A.M., Haussler D. (2002). The human genome browser at UCSC. Genome Res..

[56] Machanick P., Bailey T.L. (2011). MEME-ChIP: Motif analysis of large DNA datasets. Bioinformatics.

[57] Bailey T.L., Boden M., Buske F.A., Frith M., Grant C.E., Clementi L., Ren J., Li W.W., Noble W.S. (2009). Meme SUITE: Tools for motif discovery and searching. Nucleic Acids Res..

[58] Kim D., Pertea G., Trapnell C., Pimentel H., Kelley R., Salzberg S.L. (2013). TopHat2: Accurate alignment of transcriptomes in the presence of insertions, deletions and gene fusions. Genome Biol..

[59] Livak K.J., Schmittgen T.D. (2001). Analysis of relative gene expression data using real-time quantitative PCR and the 2(-Delta Delta C(T)) method. Methods.

[60] Mukhopadhyay A., Deplancke B., Walhout A.J., Tissenbaum H.A. (2008). Chromatin immunoprecipitation (ChIP) coupled to detection by quantitative real-time PCR to study transcription factor binding to DNA in Caenorhabditis elegans. Nat. Protoc..

[61] Perez-Riverol Y., Csordas A., Bai J., Bernal-Llinares M., Hewapathirana S., Kundu D.J., Inuganti A., Griss J., Mayer G., Eisenacher M., Perez E., Uszkoreit J., Pfeuffer J., Sachsenberg T., Yilmaz S. (2019). The PRIDE database and related tools and resources in 2019: Improving support for quantification data. Nucleic Acids Res..

