# Supplementary material for: Methyl-lysine readers PHF20 and PHF20L1 define two distinct gene expression–regulating NSL complexes
Source: J Biol Chem. 2022 Jan 14;298(3):101588. doi: 10.1016/j.jbc.2022.101588 (PMC8867114; doi:10.1016/j.jbc.2022.101588)
Supplement: Supplemental Tables S1–S2 [file mmc1.docx]

**Table S1.** Non-NSL complex proteins that were identified from mass spectrometry analysis of Flag immunoprecipitated products from U2OS over-expressing Flag-tagged PHF20 or PHF20L1 and interacted with only PHF20 or PHF20L1.

| Identified proteins | Alternative IDs | Unique Peptides | Coverage (%) | Identification Probability (%) |
| --- | --- | --- | --- | --- |
| **PHF20 Interacting Proteins** | | | | |
| General transcription factor 3C polypeptide 4 | GTF3C4 | 2 | 7.5 | 100 |
| General transcription factor 3C polypeptide | GTF3C2 | 2 | 4.4 | 100 |
| Ras-responsive element-binding protein 1 | RREB1 | 2 | 4.6 | 100 |
| Y-box-binding protein 3 | YBX3 | 2 | 12 | 100 |
| Scaffold attachment factor B1 | SAFB | 2 | 4.5 | 100 |
| Nuclear pore complex protein Nup155 | NUP155 | 3 | 3.2 | 100 |
| Melanoma-associated antigen D2 | MAGED2 | 2 | 7.8 | 100 |
| mRNA cap guanine-N7 methyltransferase | RNMT | 2 | 6.1 | 100 |
| Down syndrome cell adhesion molecule-like protein 1 | DSCAML1 | 2 | 4.4 | 100 |
| Mitochondrial ribosome-associated GTPase 1 | MTG1 | 2 | 9.3 | 100 |
| Paraspeckle component 1 | PSPC1 | 2 | 5.9 | 100 |
| Calumenin | CALU | 2 | 15 | 100 |
| T-complex protein 1 subunit zeta | CCT6A | 2 | 6.2 | 100 |
| Procollagen galactosyltransferase 1 | COLGALT1 | 2 | 3.7 | 100 |
| Rab GDP dissociation inhibitor beta | GDI2 | 3 | 10 | 100 |
| Obscurin | OBSCN | 3 | 0.665 | 100 |
| Alpha-amylase 1 | AMY1A | 4 | 10 | 100 |
| **PHF20L1 Interacting Proteins** | | | | |
| Chromodomain-helicase-DNA-binding protein 1 | CHD1 | 2 | 1.8 | 100 |
| Transcriptional repressor p66-beta | GATAD2B | 3 | 15 | 100 |
| Aconitate hydratase, mitochondrial | ACO2 | 2 | 4.9 | 100 |
| Prohibitin-2 | PHB2 | 2 | 10 | 100 |
| Solute carrier organic anion transporter family member 1B1 | SLCO1B1 | 2 | 6.5 | 100 |

**Table S2.** Sequences of short-hairpin RNAs (shRNAs) and primers used in this study.

|  | **Sequences (5’ – 3’)** |
| --- | --- |
| **Short-hairpin RNAs (shRNAs)** | |
| shPHF20 (3’-UTR) | ATTGTGCCACTGATGATAAAC |
| shPHF20-1 | TAACAGGTGTATTTCTCGG |
| shPHF20-2 | TTGTTACTTCCACAGTCGA |
| shPHF20L1 (3’-UTR) | TACGGAAATTTAGGGTATTTA |
| shPHF20L1-1 | AGTATTCCAATCTTCAGCT |
| shPHF20L1-2 | TTTCTATTAGGTCCATCCT |
| **Chromatin Immunoprecipitation (ChIP) – qPCR primers** | |
| NAGPA_TSS FP | GTCGCCATATTGGACCGGG |
| NAGPA_TSS RP | GCGCAGGTAATTCAGTCCGA |
| **Expression qRT-PCR Primers** | |
| PHF20 FP | TCGAAGCCTGCCATAAAGGTA |
| PHF20 RP | TTCATTCAACGATGTGCACAGA |
| PHF20L1 FP | AGTACATCTGCTATATCTG |
| PHF20L1 RP | CAACCACTCCTTATCATA |
| NAGPA FP | CTGGTGCTCTTTCATGCAGACG |
| NAGPA RP | ATGGCGTTGACCACGTCCTGTT |
| NUDCD3 FP | AATGGTGCTGTCCGAGAG |
| NUDCD3 RP | GAGACCTGCTTTCCCTTCA |
| LAMP1 FP | AGTCGGCAATTCCTACAAG |
| LAMP1 RP | CAGACACTCCTCCACAGA |
| VAMP3 FP | CGCAGCCAAGTTGAAGAGG |
| VAMP3 RP | CCACACGATGATGATGATGATGAA |
| PIGT FP | CAGGACAACGAGACATTAGAG |
| PIGT RP | CAAGCAAGTCATAGATGGCATA |
| RPLP0 FP | TTCATTGTGGGAGCAGAC |
| RPLP0 RP | CAGCAGTTTCTCCAGAGC |
| *Phf20* FP | TGAGAAATACACCTGCTA |
| *Phf20* RP | CTCCTTGTCATACCAGTA |
| *Phf20l1* FP | ATGAATCTGGAGAGTCTT |
| *Phf20l1* RP | CCTTGGCTTGTATTAACT |
| *Nagpa* FP | GACGAGACACAGGAGACA |
| *Nagpa RP* | CAGCATGGAAGAGGATAAGC |
| *Nudcd3* FP | CTCCAGGAACAGTTAGCA |
| *Nudcd3* RP | CAGGTCGGTGTAGTCTTG |
| *Lamp1* FP | GCAACTGAATATCACCTACCT |
| *Lamp1* RP | CTCTGTTCTTGTTCTCCACTT |
| *Vamp3* FP | AAGTGCTGCCAAGTTGAAGAGAA |
| *Vamp3* RP | CACACCACACGATGATGATGATGA |
| *Pigt* FP | GCTCAGCAATGTCCTCTC |
| *Pigt* RP | GAAGTAGTCGTCAGTGTCAT |
| *Rplp0* FP | AATCTCCAGAGGCACCATTGA |
| *Rplp0* RP | GTTCAGCATGTTCAGCAGTGT |
