# Supplementary figures and images for: Methyl-lysine readers PHF20 and PHF20L1 define two distinct gene expression–regulating NSL complexes
Source: J Biol Chem. 2022 Jan 14;298(3):101588. doi: 10.1016/j.jbc.2022.101588 (PMC8867114; doi:10.1016/j.jbc.2022.101588)

Figure S2, Van et al

**A**

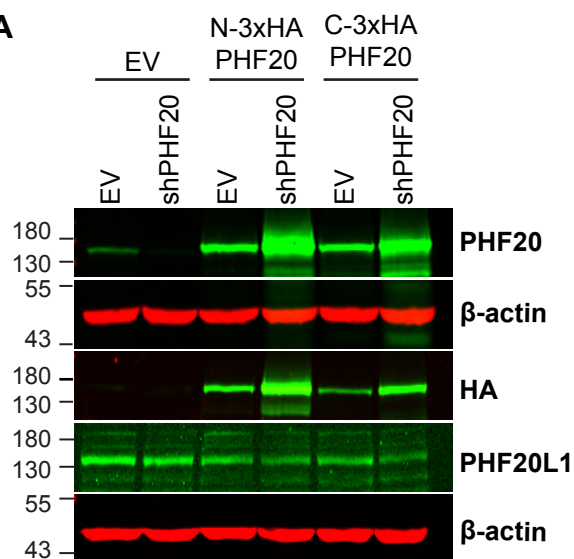

**B**

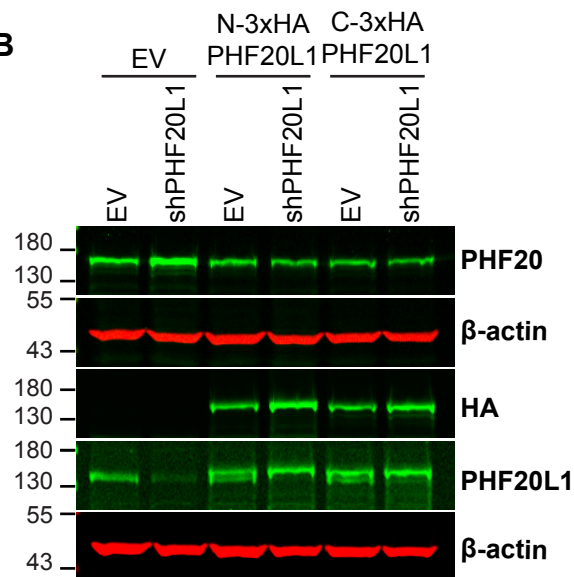

**C**

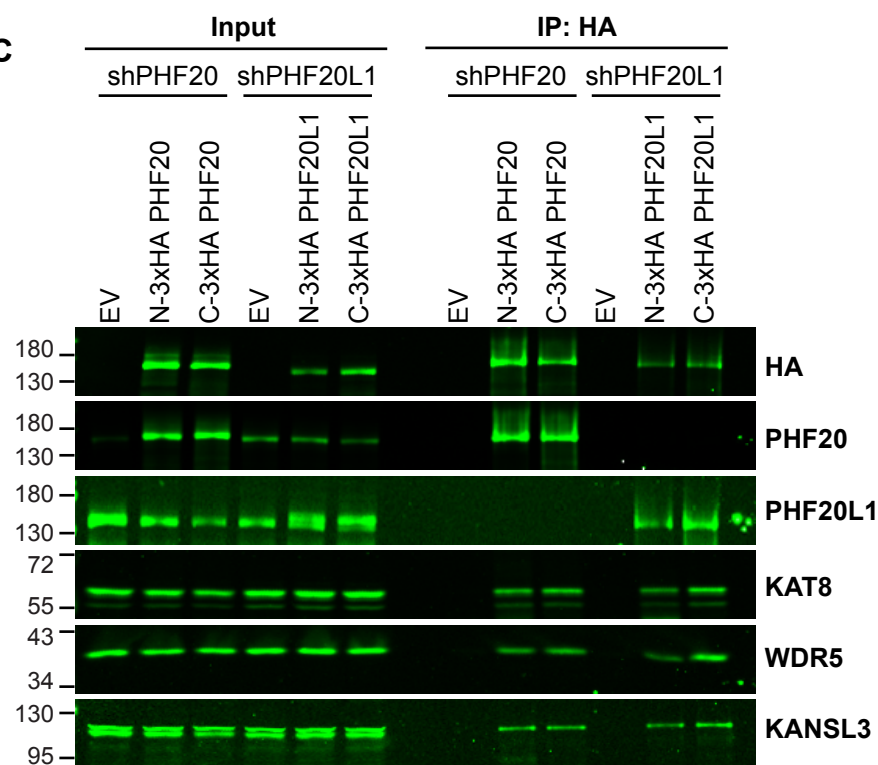

Supplement: Supplemental Figure S2 [file mmc3.pdf]

Figure S3, Van et al

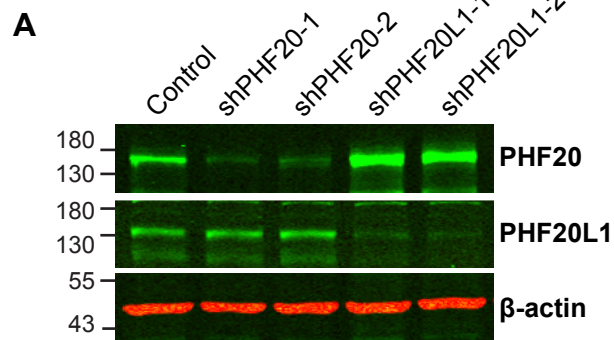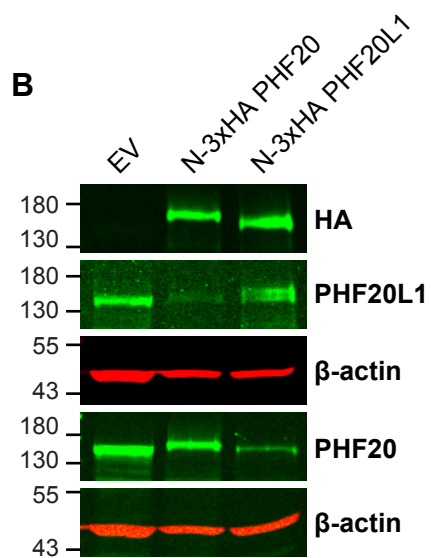

Supplement: Supplemental Figure S3 [file mmc4.pdf]

Figure S4, Van et al

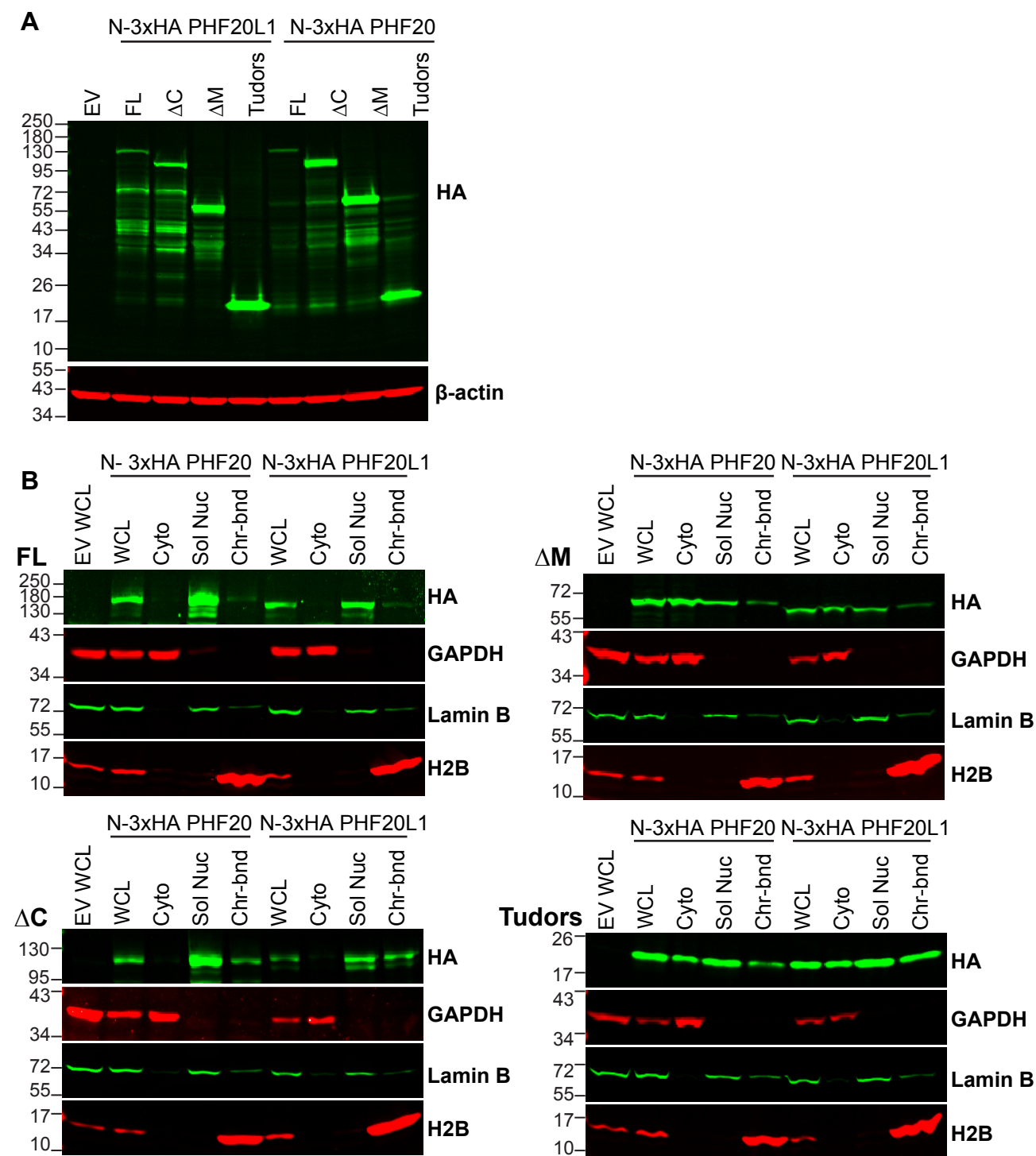

Supplement: Supplemental Figure S4 [file mmc5.pdf]

Figure S5, Van et al

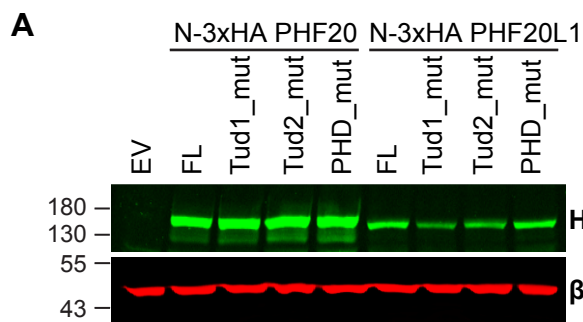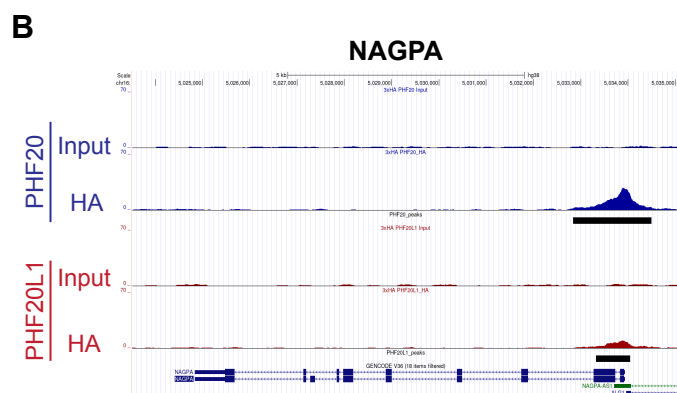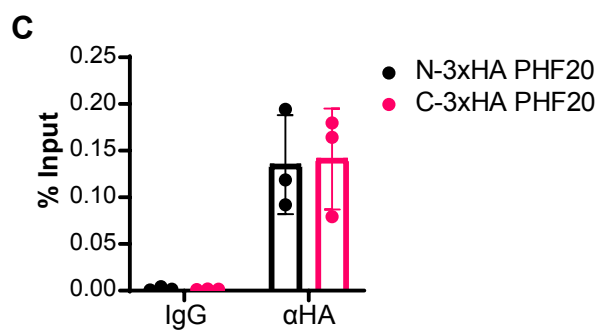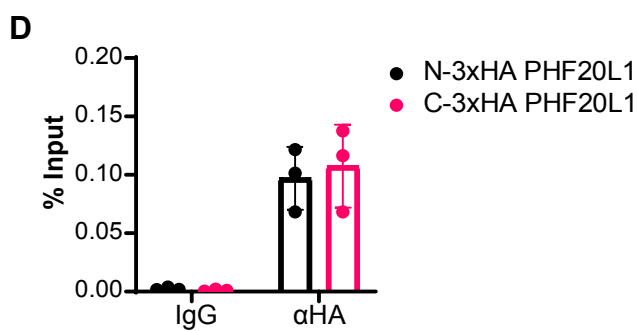

Supplement: Supplemental Figure S5 [file mmc6.pdf]

Figure S6, Van et al

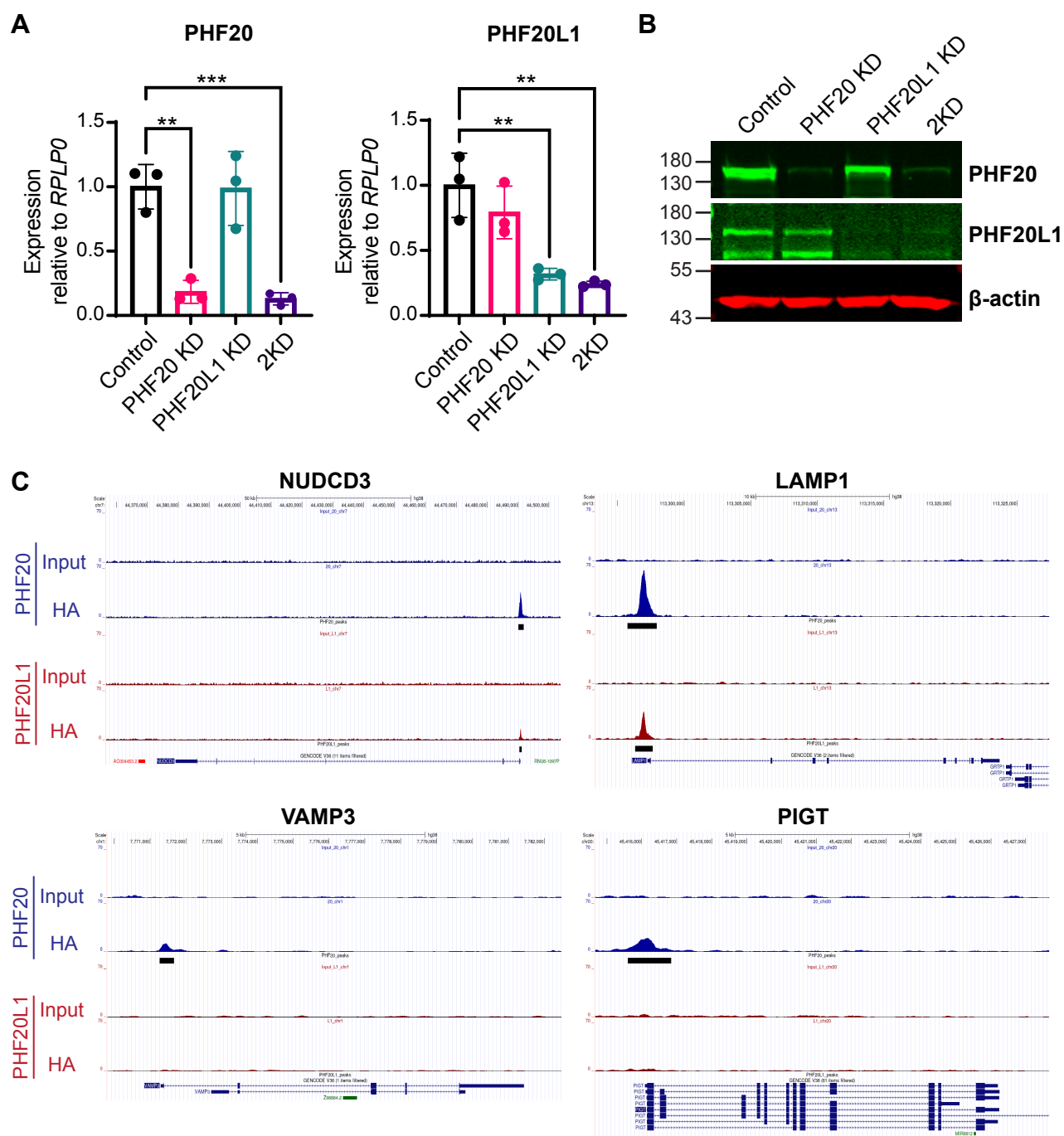

Supplement: Supplemental Figure S6 [file mmc7.pdf]

Figure S7, Van et al

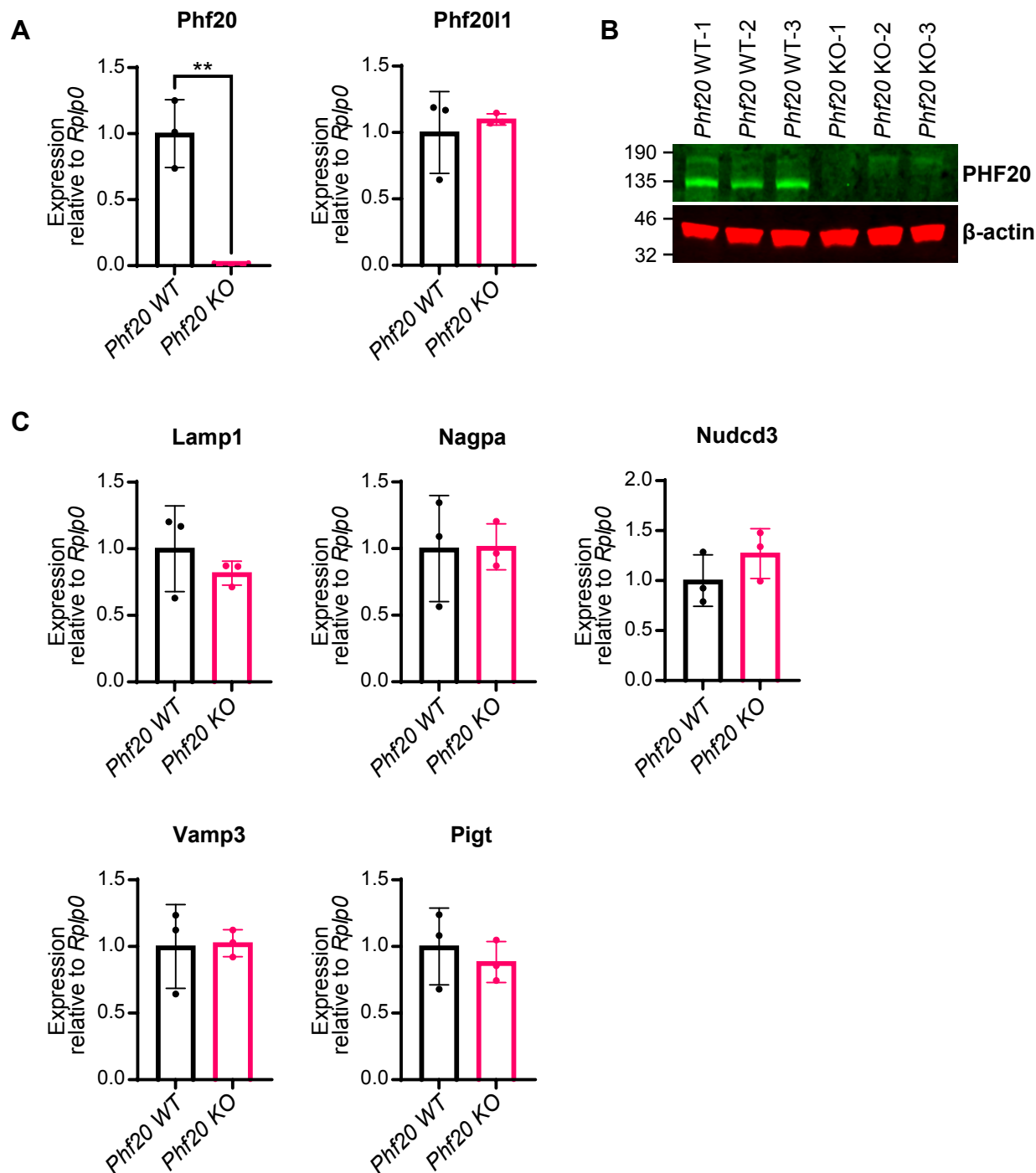

Supplement: Supplemental Figure S7 [file mmc8.pdf]

Figure S8, Van et al

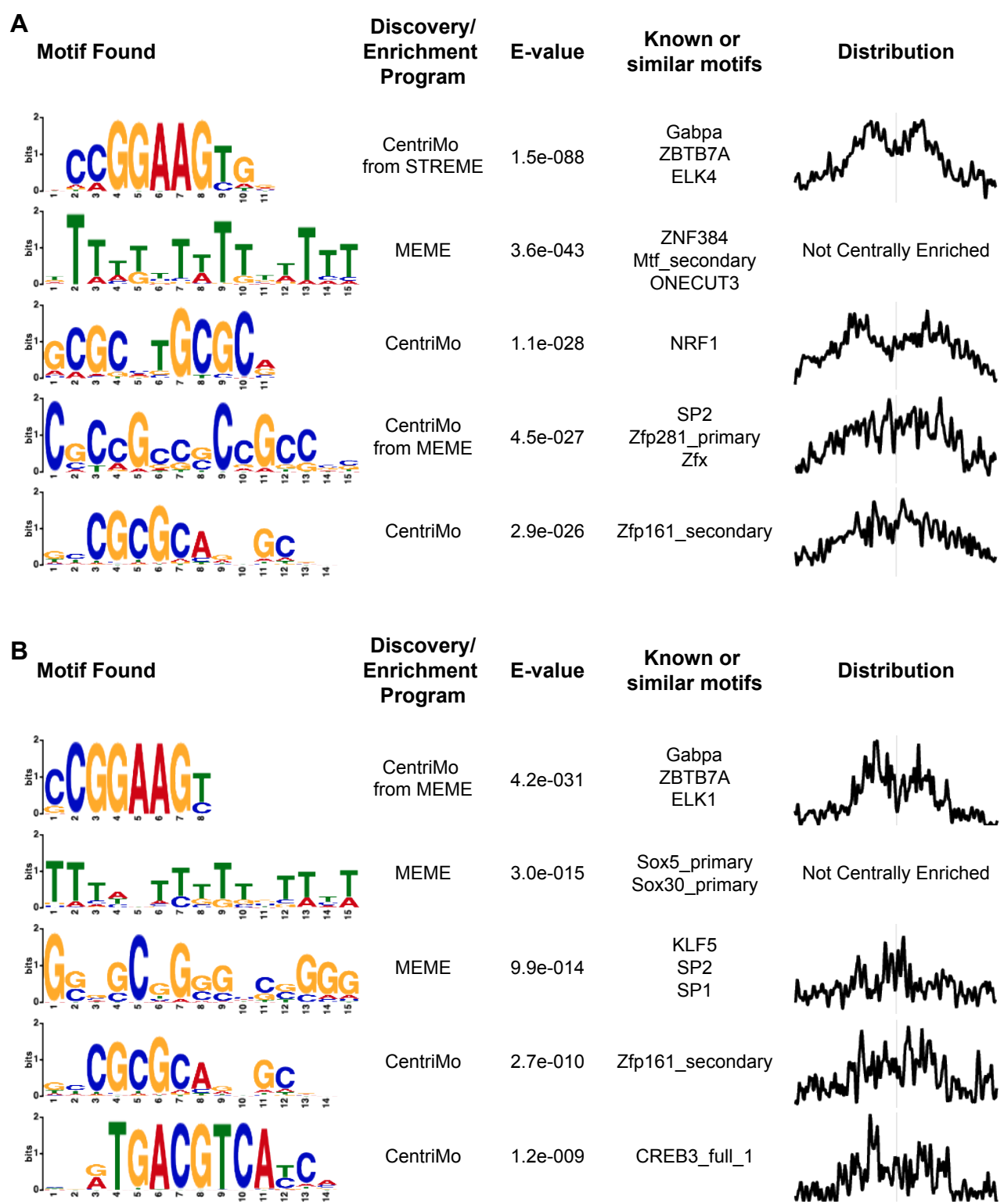

Supplement: Supplemental Figure S8 [file mmc9.pdf]

Figure S9, Van et al

**A**

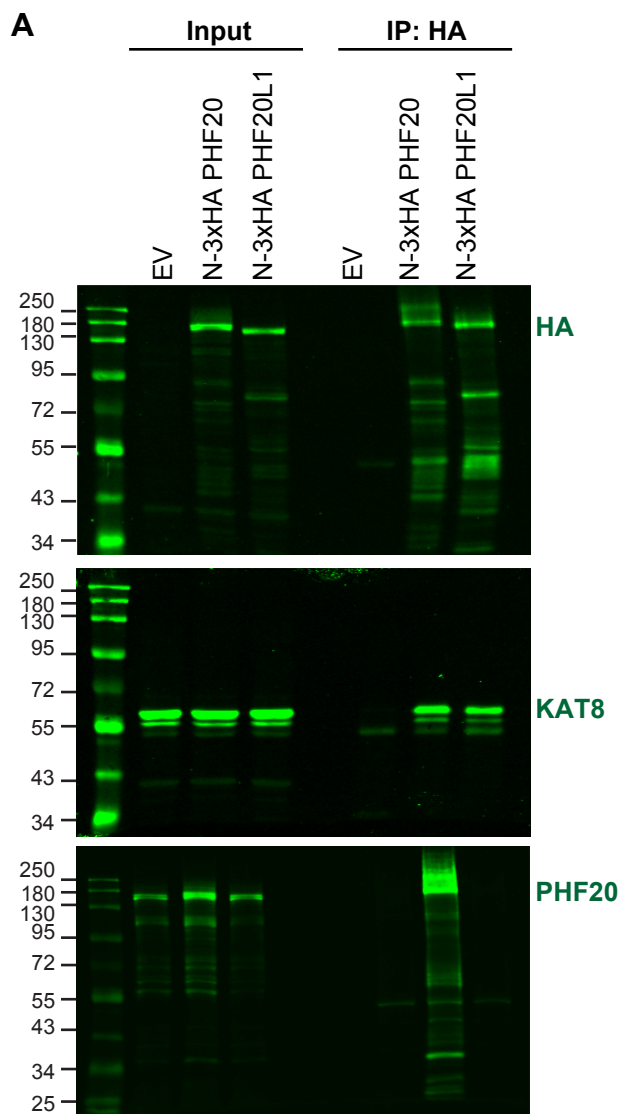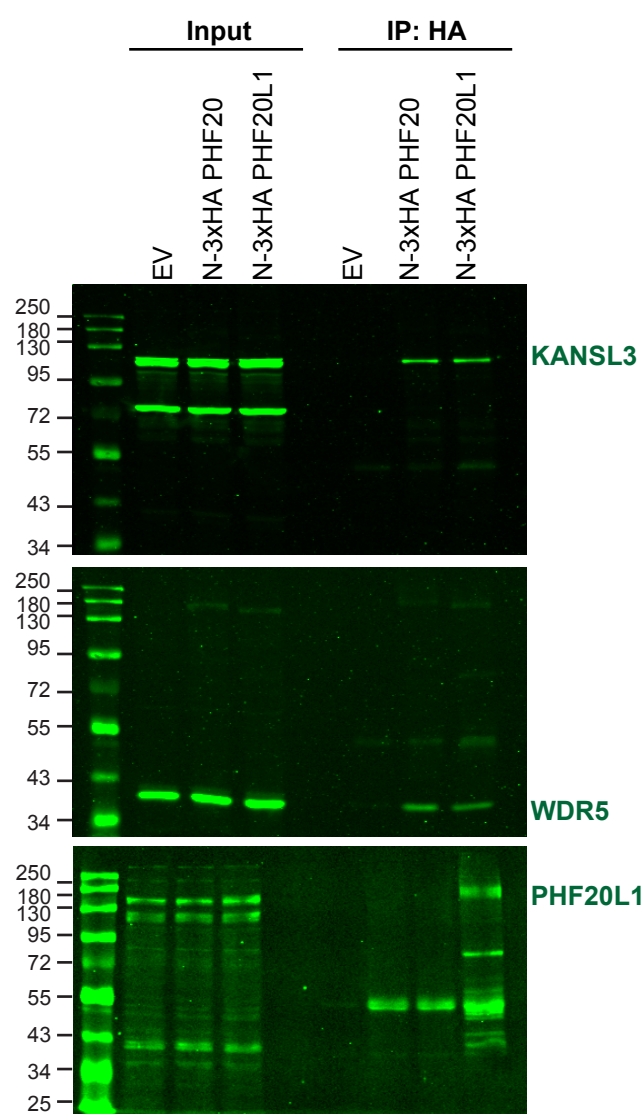

Supplement: Supplemental Figure S9.1 [file mmc10.pdf]

| B           | Input       | IP: MOF    |
|-------------|-------------|------------|
|             | Control     | Beads only |
| shPHF20-1   | Control     |            |
| shPHF20-2   | shPHF20-1   |            |
| shPHF20L1-1 | shPHF20-2   |            |
| shPHF20L1-2 | shPHF20L1-1 |            |
|             | shPHF20L1-2 |            |

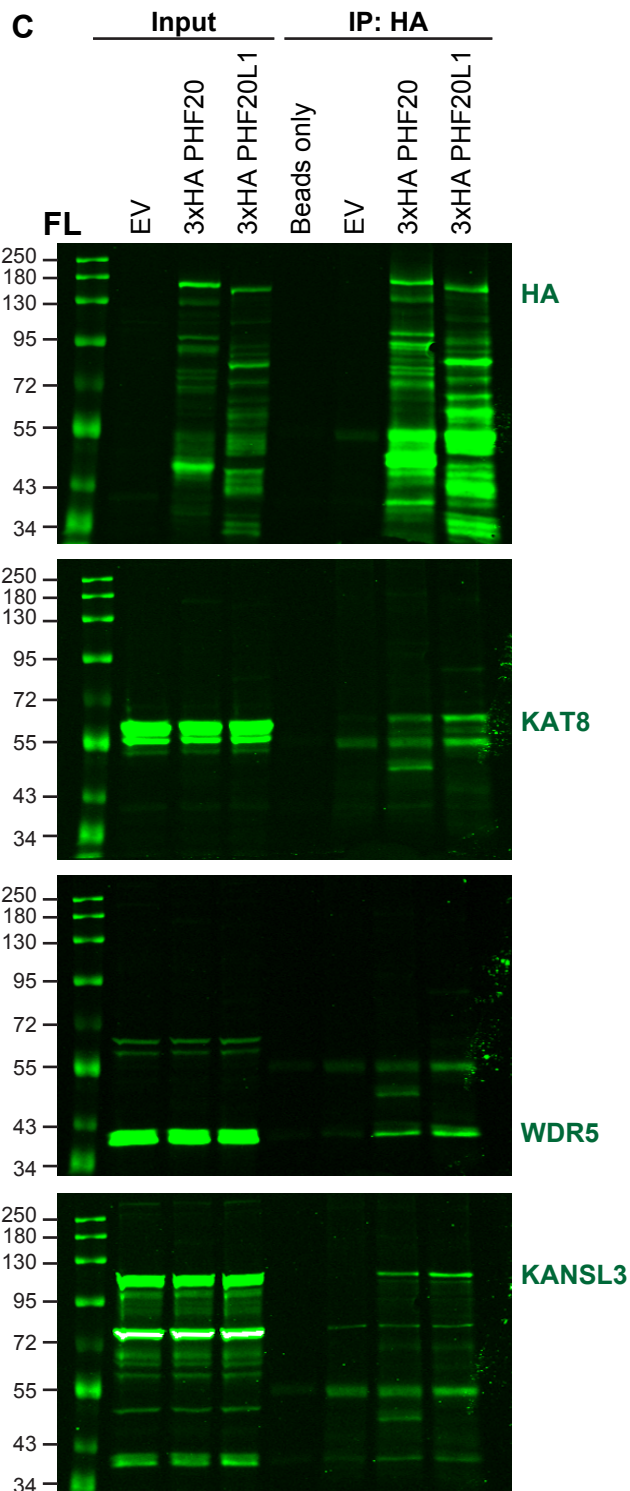

Supplement: Supplemental Figure S9.2 [file mmc11.pdf]

Figure S9, Van et al (cont.)

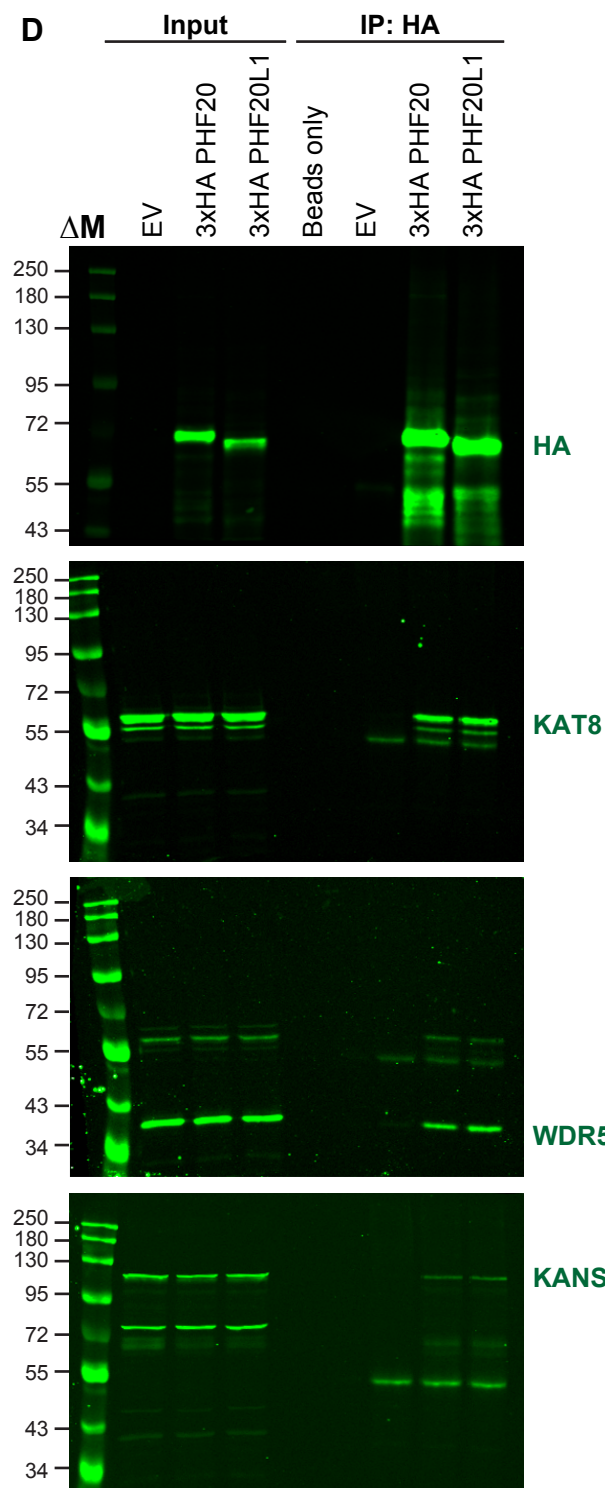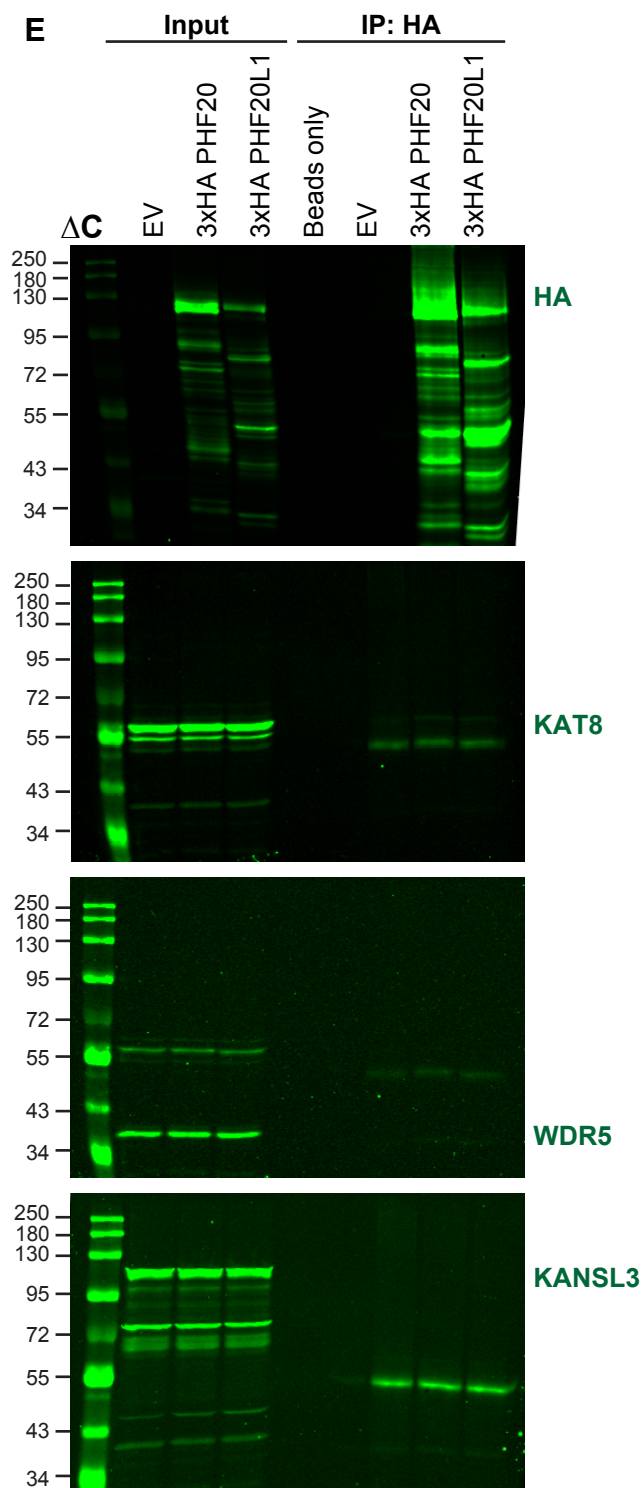

Supplement: Supplemental Figure S9.3 [file mmc12.pdf]

Figure S10, Van et al

**A**

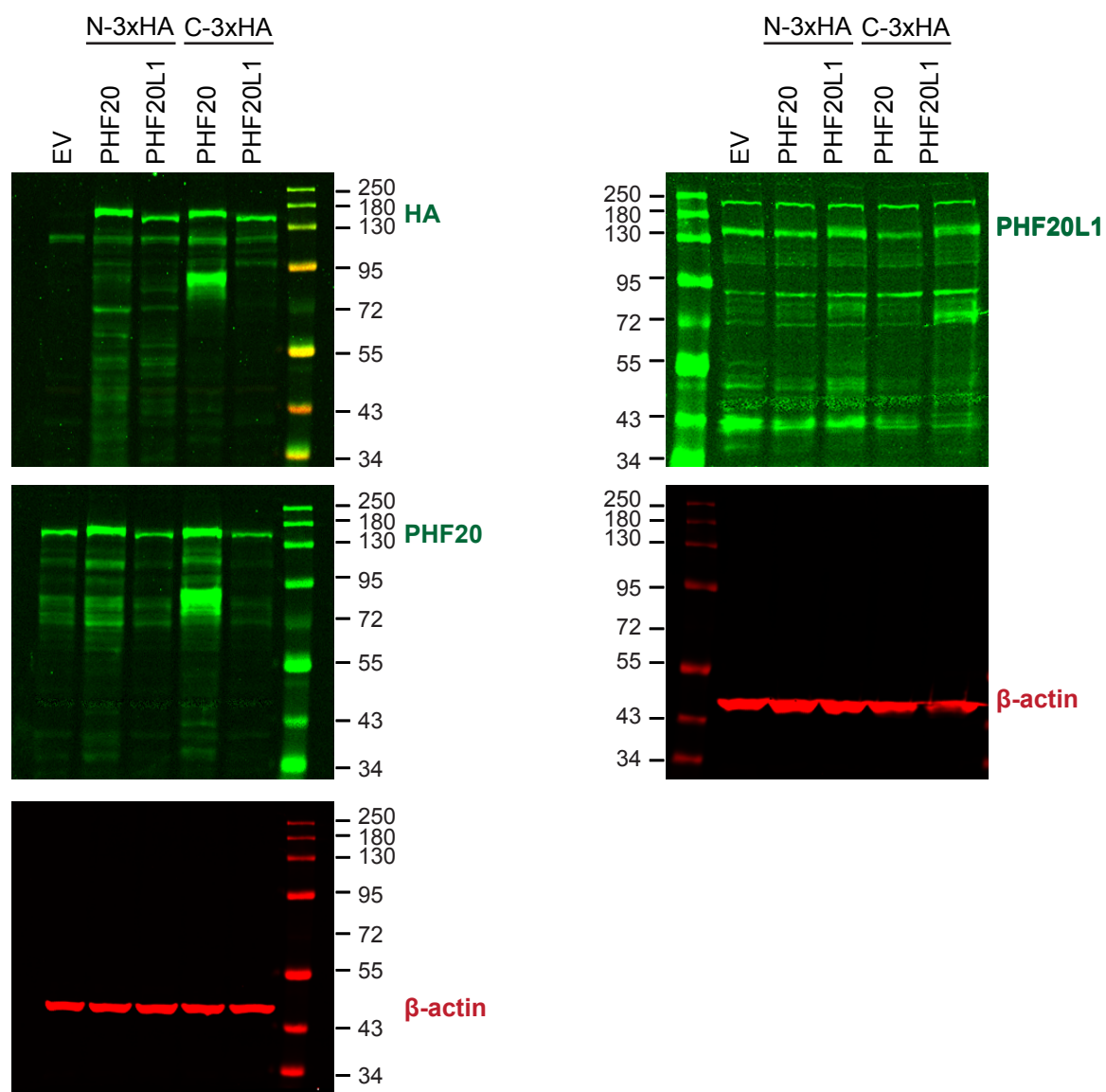

Supplement: Supplemental Figure S10.1 [file mmc14.pdf]

Figure S10, Van et al (cont.)

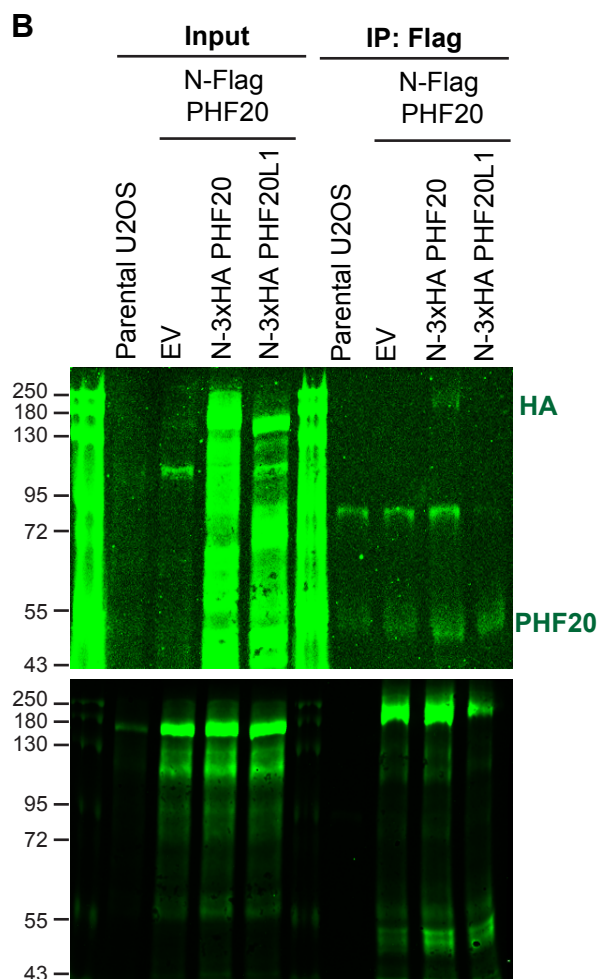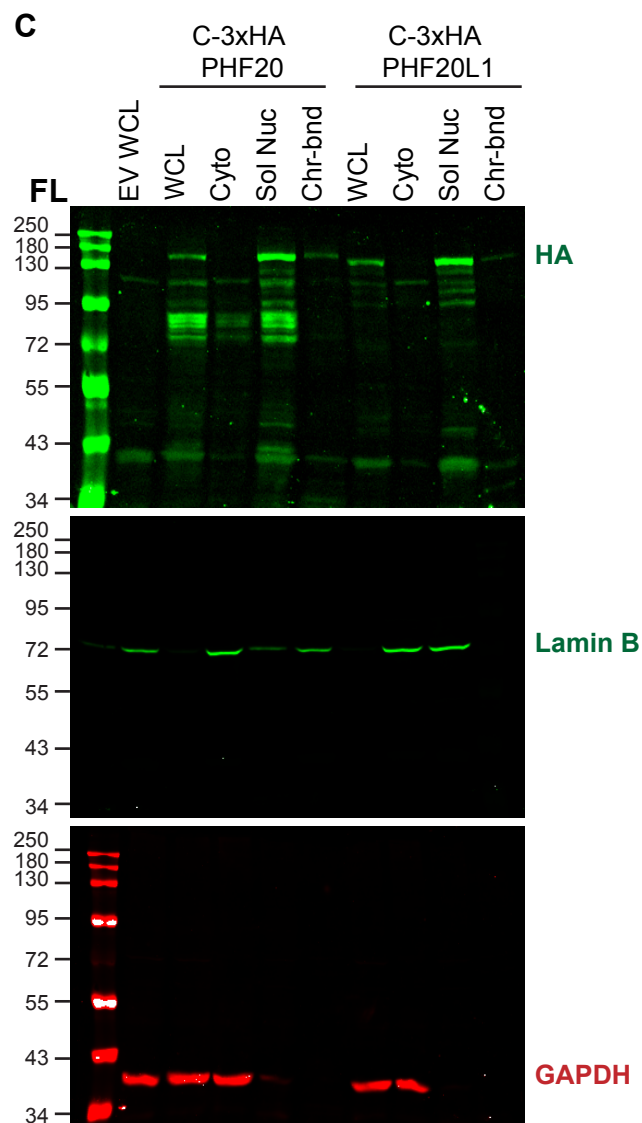

Supplement: Supplemental Figure S10.2 [file mmc15.pdf]

Figure S10, Van et al (cont.)

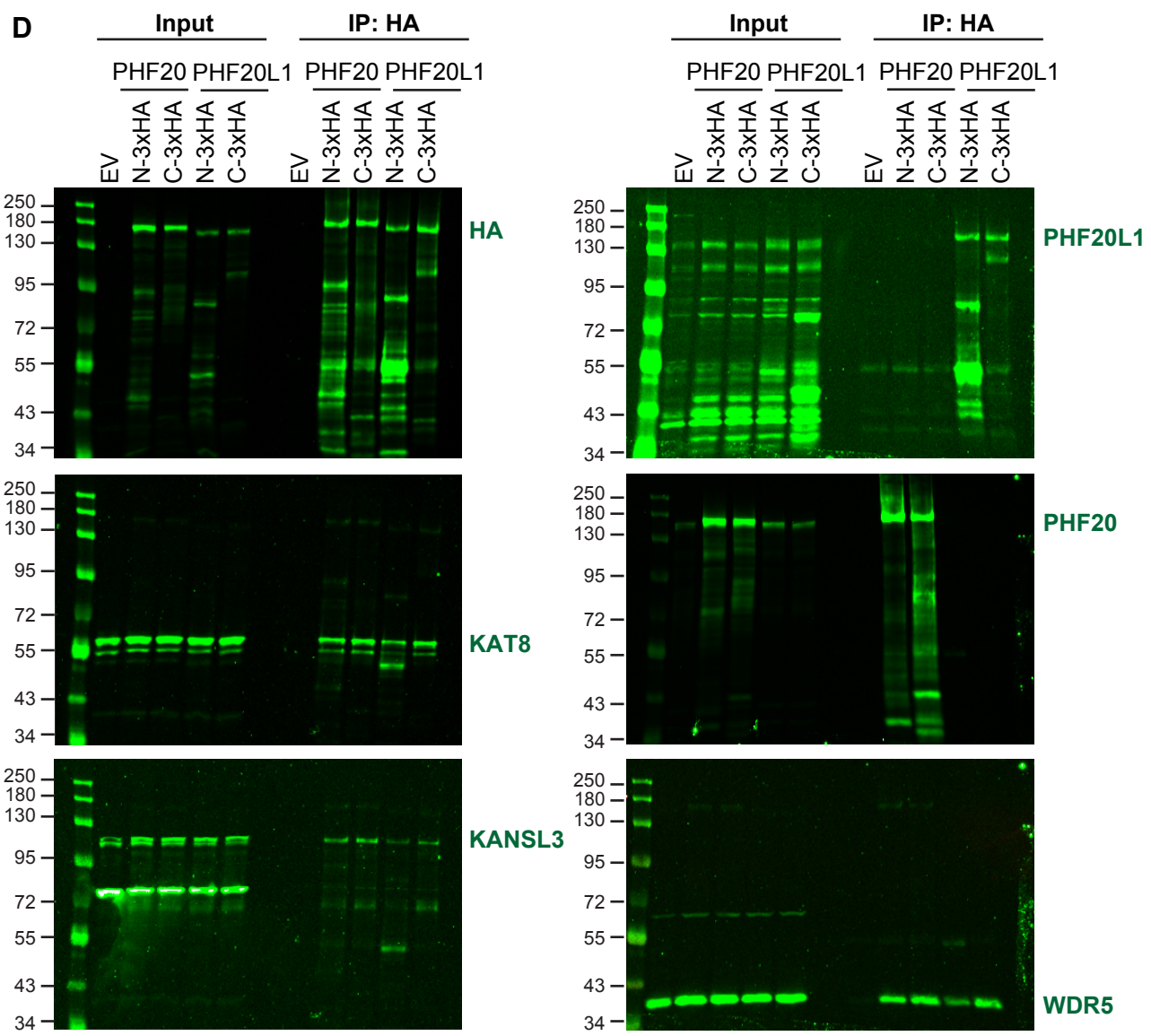

Supplement: Supplemental Figure S10.3 [file mmc16.pdf]

Figure S10, Van et al (cont.)

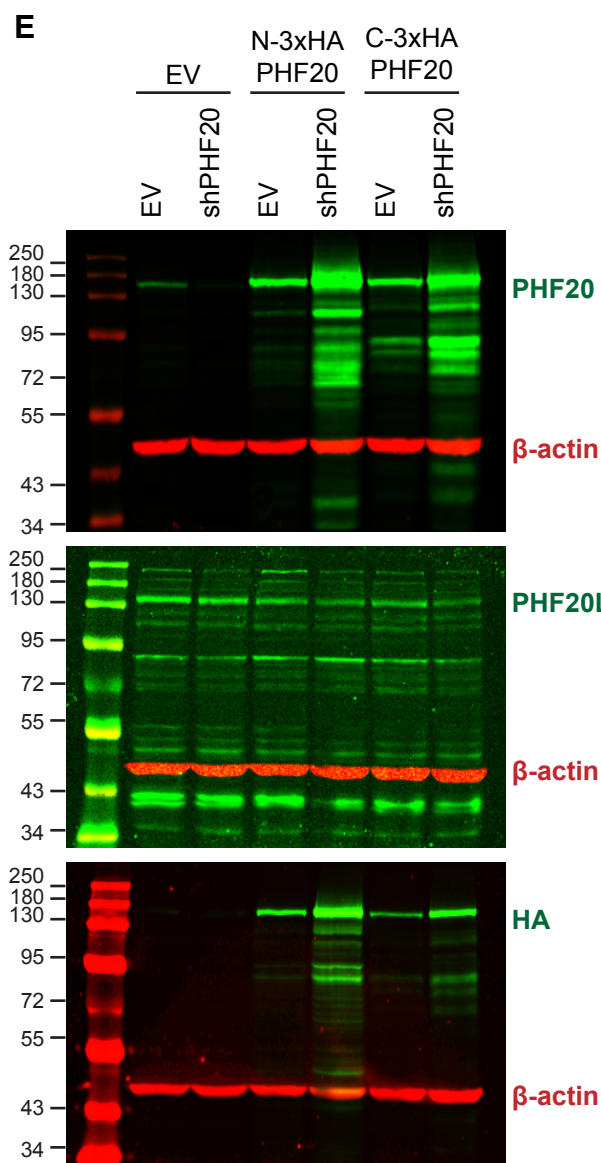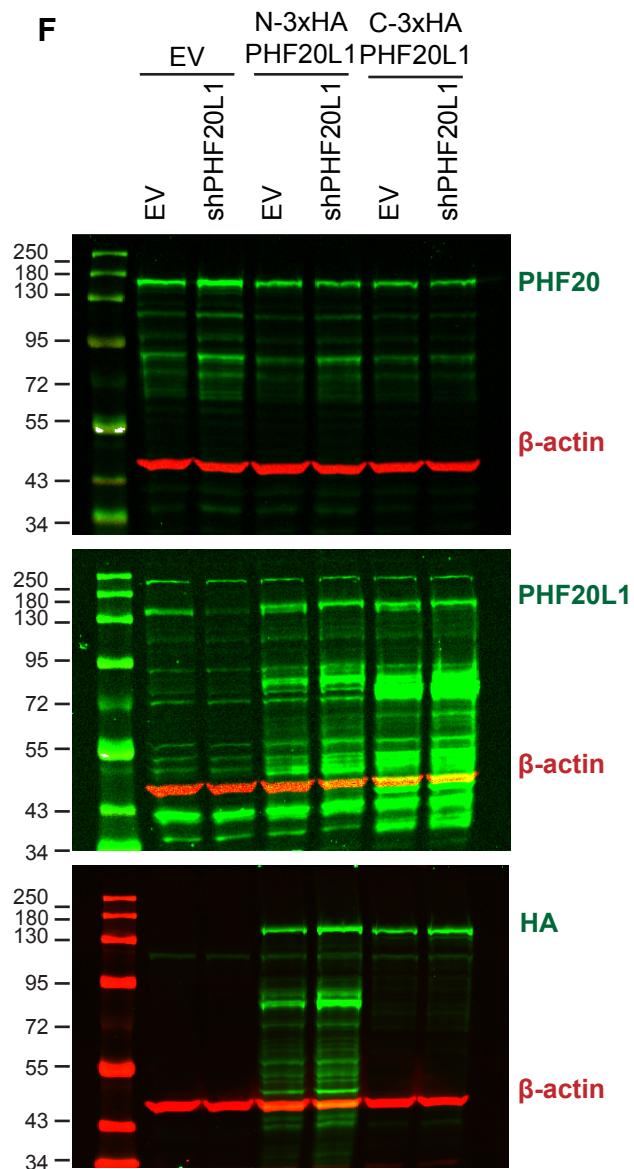

Supplement: Supplemental Figure S10.4 [file mmc17.pdf]

Figure S10, Van et al (cont.)

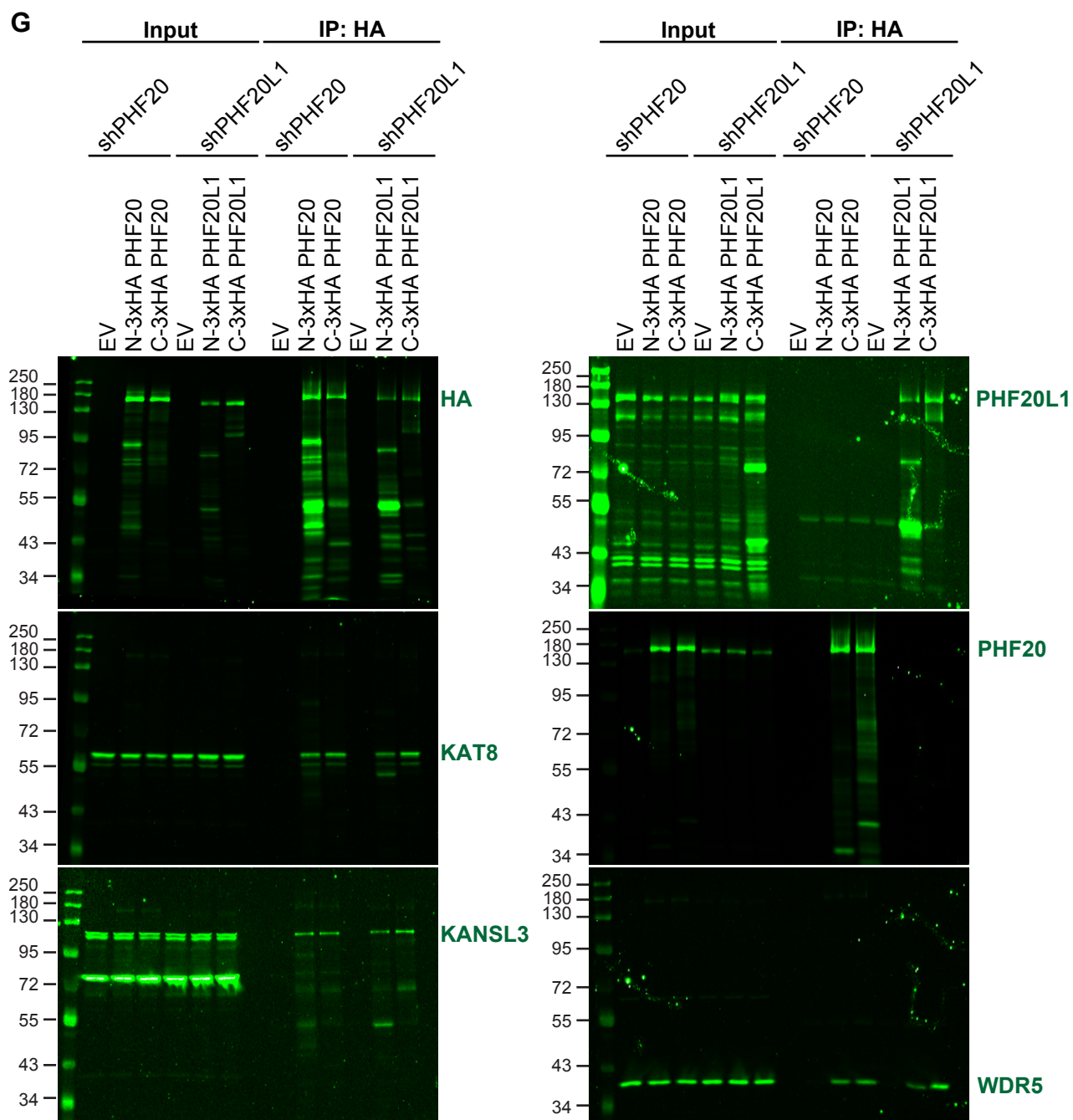

Supplement: Supplemental Figure S10.5 [file mmc18.pdf]

**H**

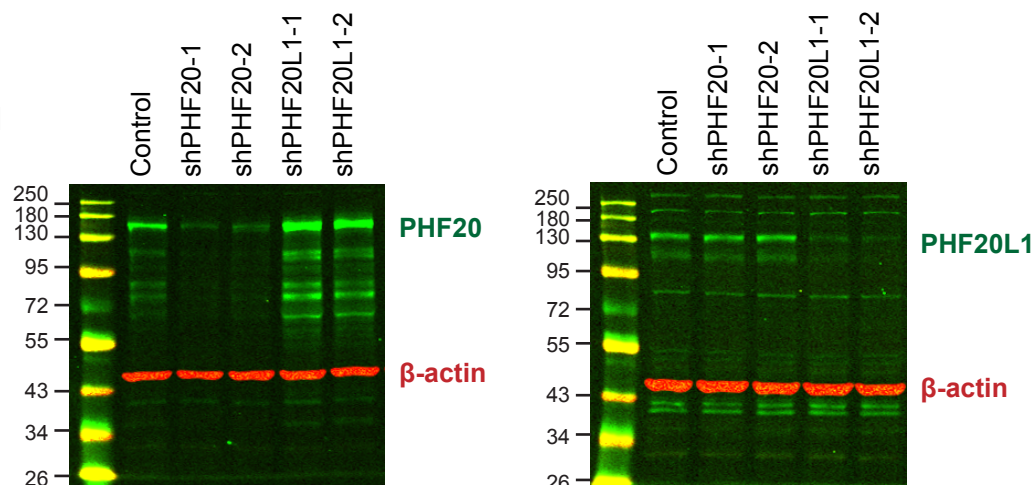

**I**

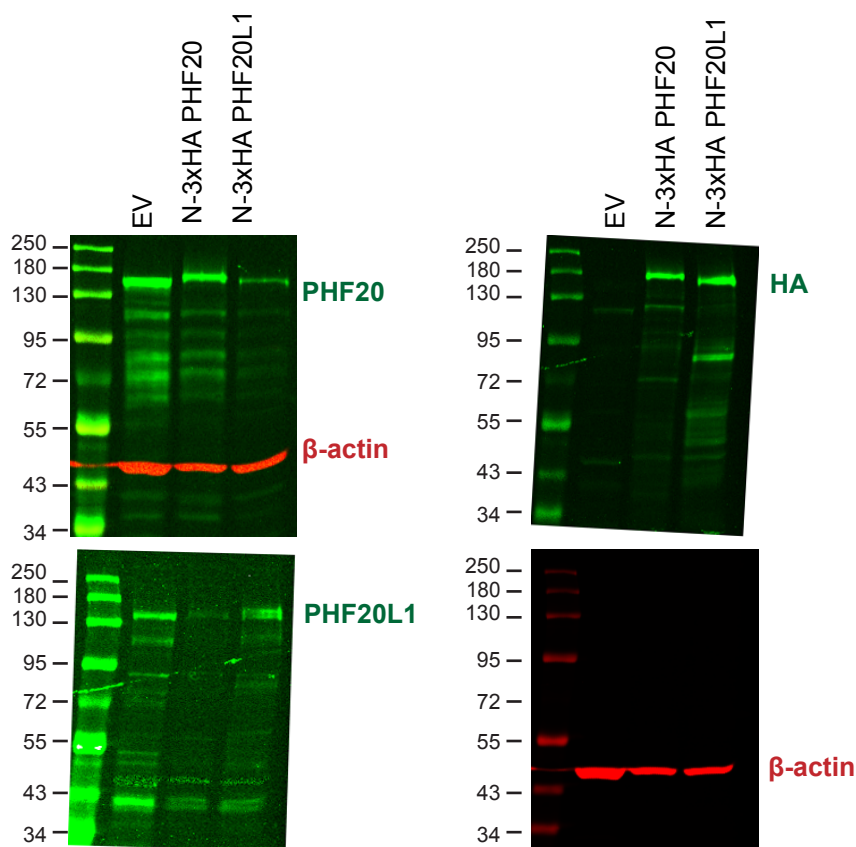

Supplement: Supplemental Figure S10.6 [file mmc19.pdf]

Figure S10, Van et al (cont.)

**J**

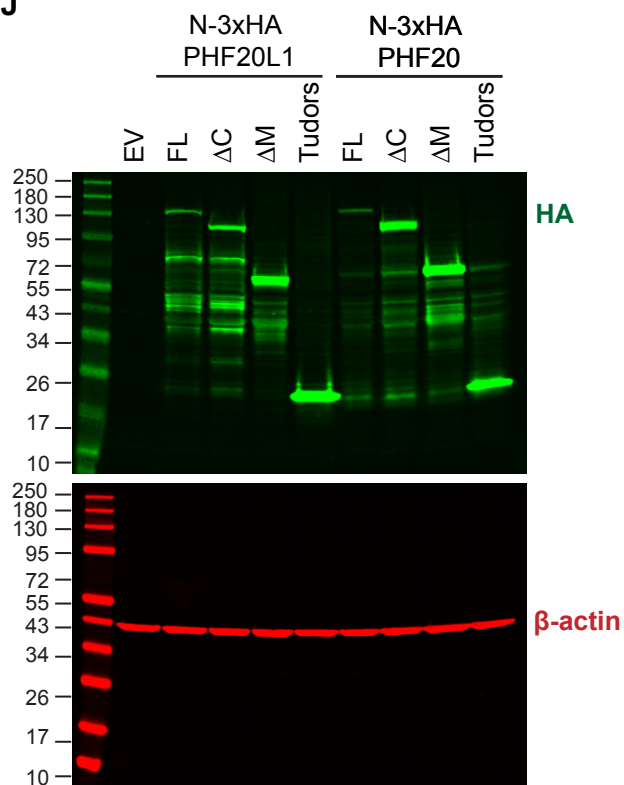

**K**

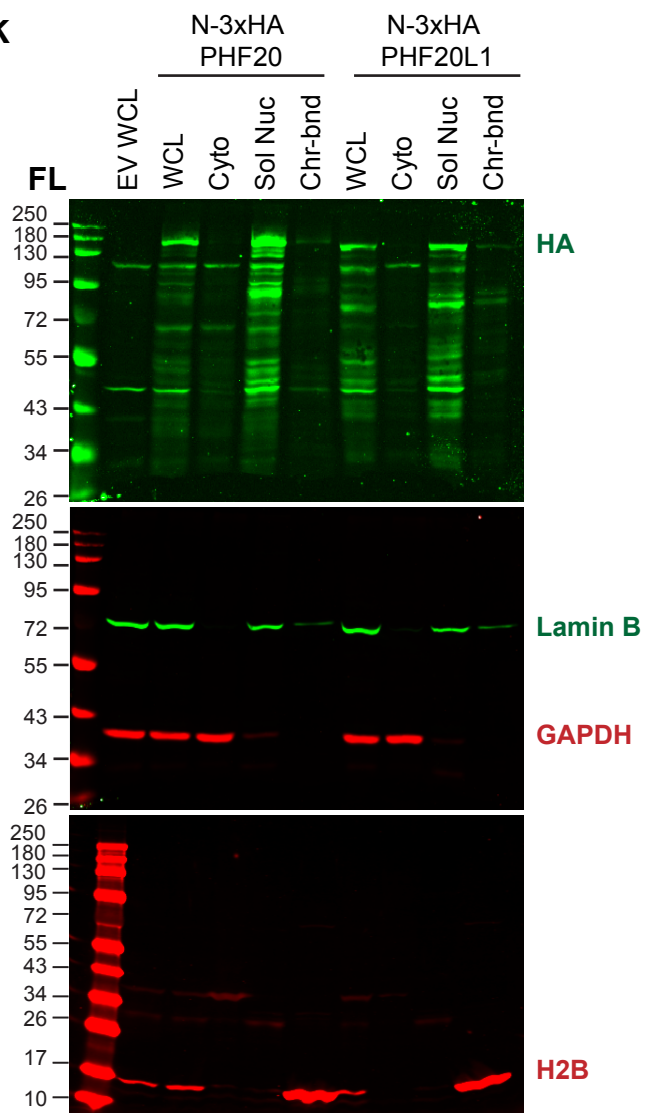

Supplement: Supplemental Figure S10.7 [file mmc20.pdf]

Figure S10, Van et al (cont.)

### K (cont.)

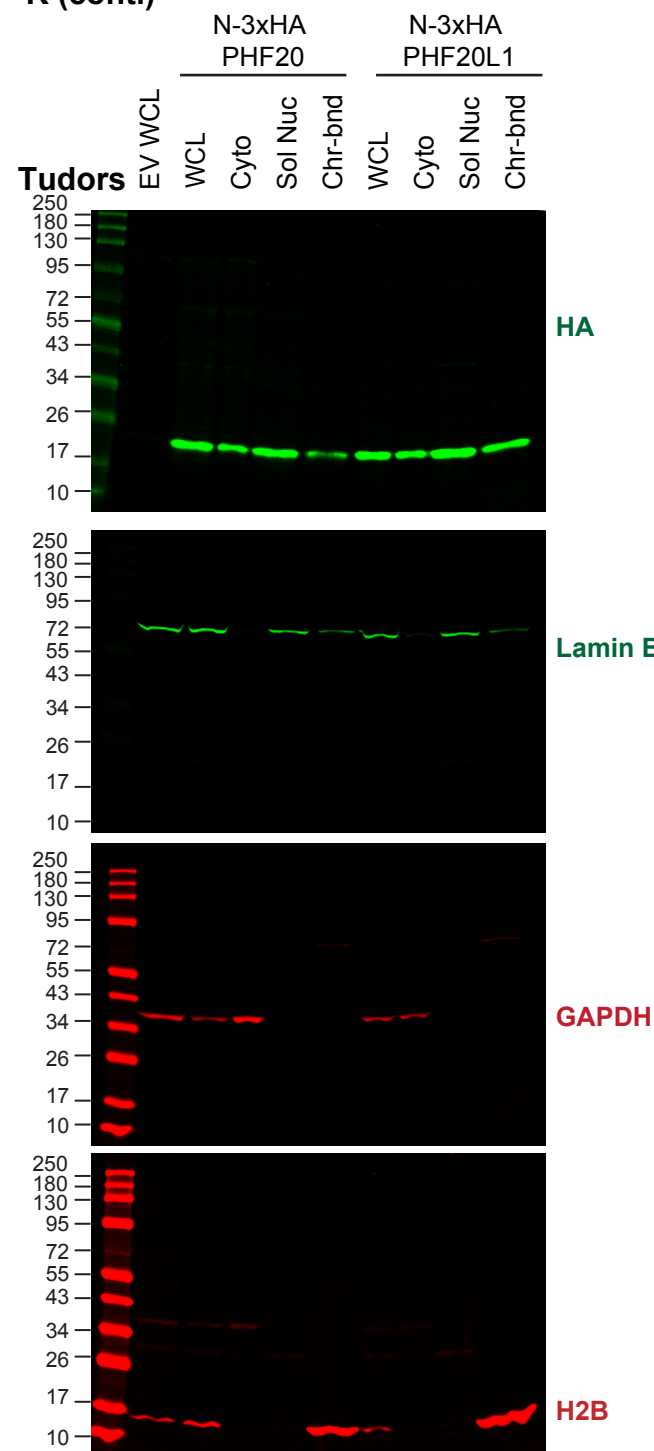

### L

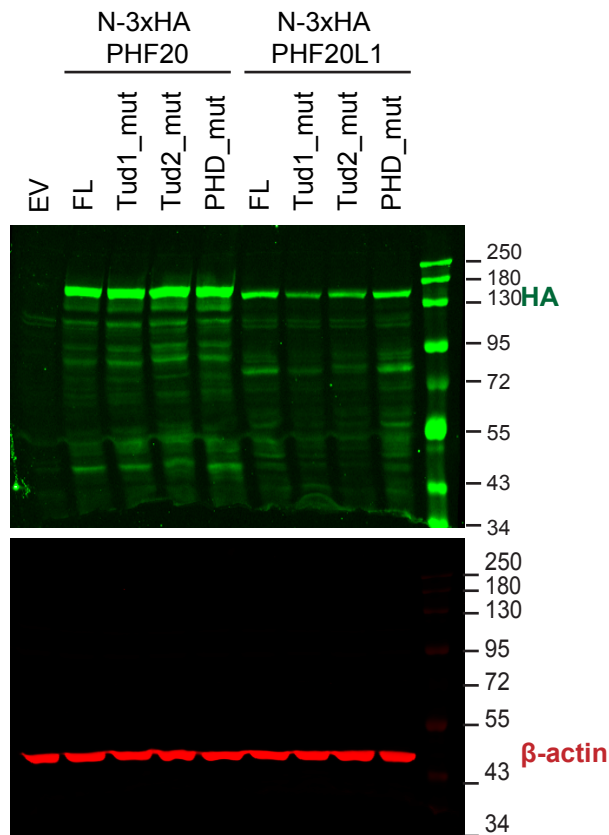

Supplement: Supplemental Figure S10.9 [file mmc22.pdf]

Figure S10, Van et al (cont.)

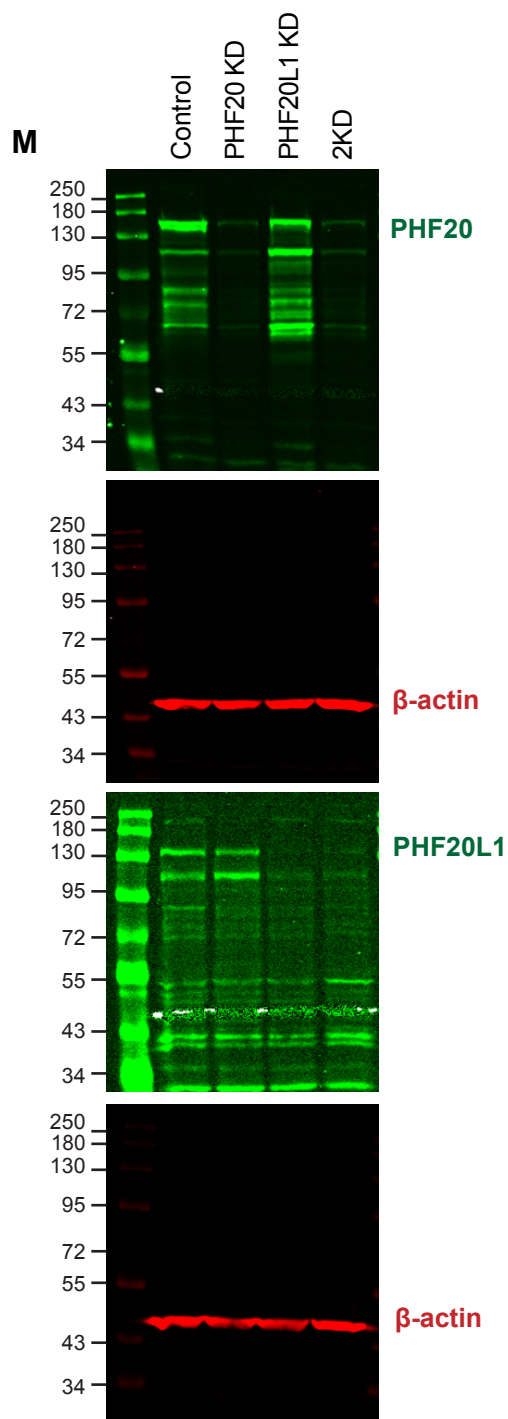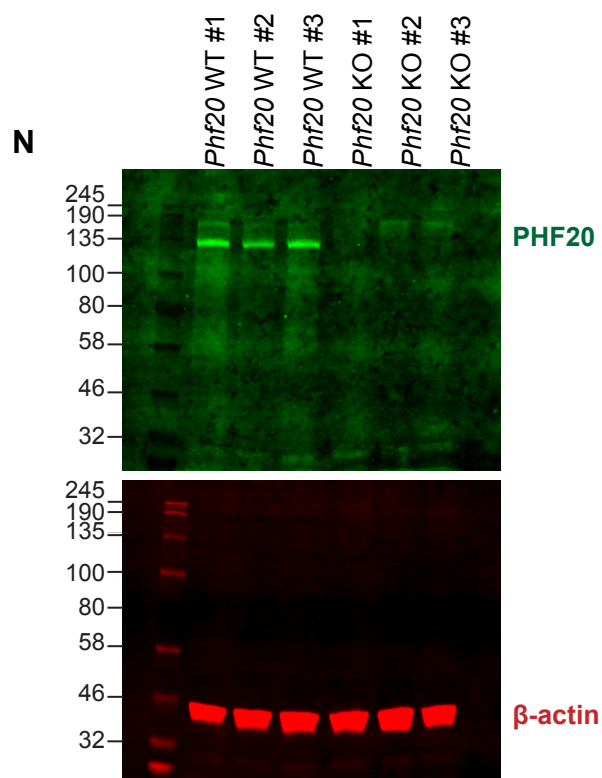

Supplement: Supplemental Figure S10.10 [file mmc23.pdf]
