# Supplementary material for: Methyl-lysine readers PHF20 and PHF20L1 define two distinct gene expression–regulating NSL complexes
Source: J Biol Chem. 2022 Jan 14;298(3):101588. doi: 10.1016/j.jbc.2022.101588 (PMC8867114; doi:10.1016/j.jbc.2022.101588)

Figure S10, Van et al (cont.)

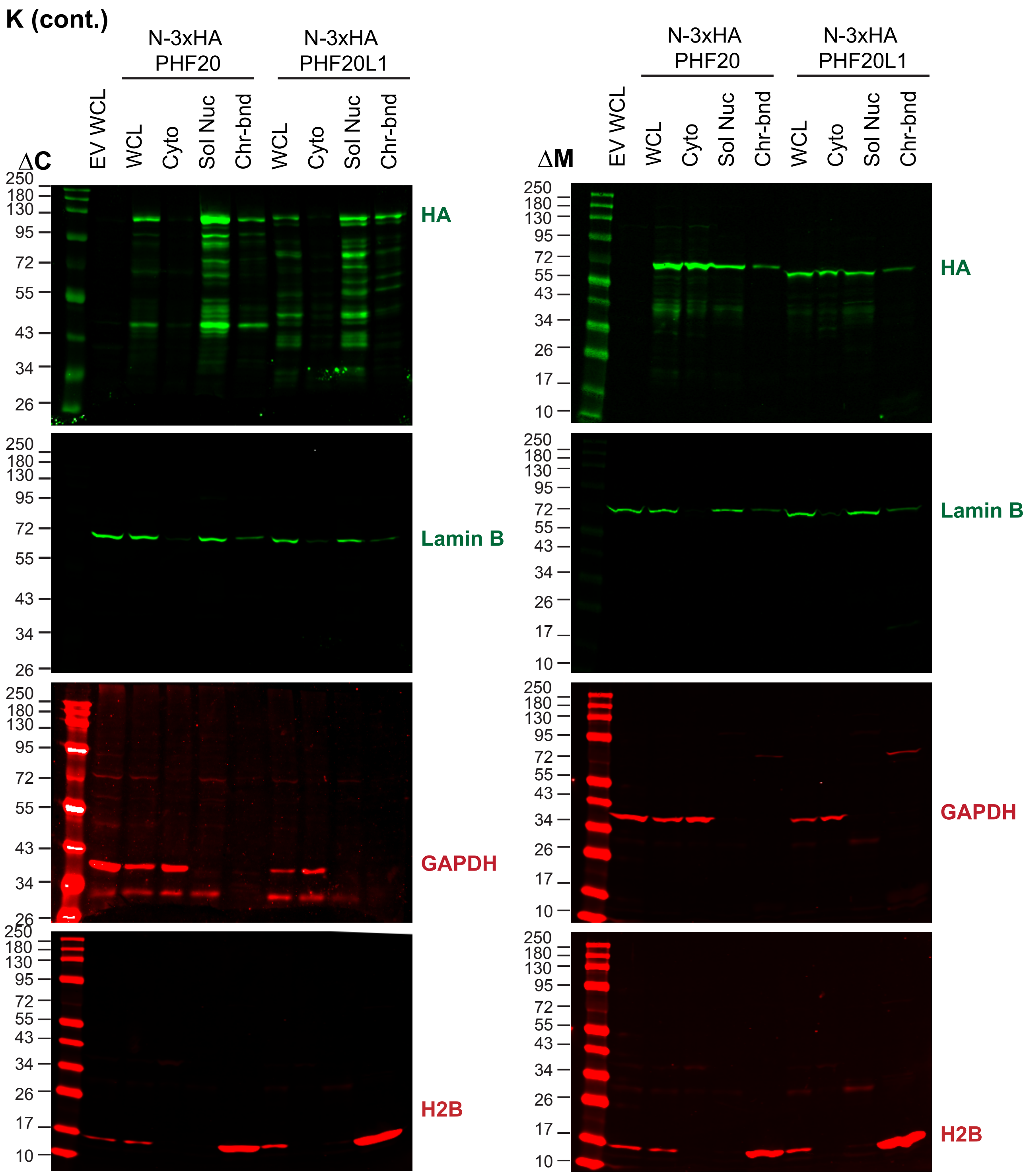

Supplement: Supplemental Figure S10.8 [file mmc21.pdf]
